# Species Diversity and Molecular Phylogeny of *Cyanosporus* (Polyporales, Basidiomycota)

**DOI:** 10.3389/fmicb.2021.631166

**Published:** 2021-02-04

**Authors:** Shun Liu, Lu-Lu Shen, Yan Wang, Tai-Min Xu, Genevieve Gates, Bao-Kai Cui

**Affiliations:** ^1^Beijing Advanced Innovation Center for Tree Breeding by Molecular Design, Beijing Forestry University, Beijing, China; ^2^School of Ecology and Nature Conservation, Institute of Microbiology, Beijing Forestry University, Beijing, China; ^3^Yichang Academy of Agricultural Science, Yichang, China; ^4^Tasmanian Institute of Agriculture, Hobart, TAS, Australia

**Keywords:** brown-rot fungi, multi-loci phylogeny, new species, *Postia caesia* complex, species identification

## Abstract

*Cyanosporus* is a cosmopolitan brown-rot fungal genus, recognizable by blue-tinted basidiocarps. Species in this genus were usually treated as belonging to the *Postia caesia* complex, however, recent phylogenetic analyses showed that this complex represents an independent genus. During further studies on *Cyanosporus*, five new species were discovered based on morphological features and molecular data. Phylogenetic analyses of *Cyanosporus* were conducted using the internal transcribed spacer (ITS) regions, the large subunit of nuclear ribosomal RNA gene (nLSU), the small subunit of nuclear ribosomal RNA gene (nSSU), the small subunit of mitochondrial rRNA gene (mtSSU), the largest subunit of RNA polymerase II (RPB1), the second largest subunit of RNA polymerase II (RPB2), and the translation elongation factor 1-α gene (TEF); illustrated descriptions of the new species are provided. In addition, fifteen species previously belonging to the *Postia caesia* complex are transferred to *Cyanosporus* and proposed as new combinations.

## Introduction

*Boletus caesius* Schrad. was described based on material from Germany (Schrader, 1794), and this name was subsequently sanctioned by Fries (1821), who considered *B. coeruleus* Schumach. a synonym of *Polyporus caesius* (Schrad.) Fr. In 1881 Karsten transferred *Boletus caesius* to *Postia* Fr. as *Postia caesia* (Schrad.) P. Karst. (Karsten, 1881). Murrill (1907) transferred this species to *Tyromyces* P. Karst. as *T. caesius* (Schrad.) Murrill and later McGinty (1909) proposed a new monotypic genus *Cyanosporus* McGinty for *Polyporus caesius*, based on its cyanophilous basidiospores, but *Cyanosporus caesius* was not widely used in subsequent studies (Donk, 1960; Jahn, 1963; Lowe, 1975), while *Tyromyces caesius* was commonly used. Later, *Postia caesia* was widely used (Papp, 2014). David (1974, 1980) described another two species: *P. luteocaesia* (A. David) Jülich and *P. subcaesia* (A. David) Jülich from Europe besides *Postia caesia* (Schrad.) P. Karst. Jahn (1979) noted that David’s *P. subcaesia* included many forms and introduced *P. subcaesia* “f. minor,” which was described as *P. alni* Niemelä & Vampola by Niemelä et al. (2001). Subsequently, Pieri and Rivoire (2005) introduced the fifth European species, *P. mediterraneocaesia* M. Pieri & B. Rivoire in the *Postia caesia* complex. Papp (2014) provided a detailed nomenclatural review on the *Postia caesia* complex and proposed the subgenus *Postia* subg. *Cyanosporus* (McGinty) V. Papp for this complex which included five species viz., *P. alni*, *P. caesia*, *P. luteocaesia*, *P. mediterraneocaesia* and *P. subcaesia*. Miettinen et al. (2018) focused on the taxonomy of the *Postia caesia* complex based on morphological features and molecular evidence and increased the species number of the *Postia caesia* complex from 10 to 24. In their study, they only focused on the species concept of the *Postia caesia* complex, the taxonomic status of this complex among *Postia* and related genera are not mentioned.

Shen et al. (2019) carried out a comprehensive study on *Postia* and related genera and confirmed that the genus *Cyanosporus* is an independent genus rather than subgenus which containing 12 accepted species including seven new species of the *Postia caesia* complex. Furthermore, phylogenetic analyses showed that *Cyanosporus* belongs to the antrodia clade (Shen et al., 2019). Morphologically, *Cyanosporus* differs from other related genera by its bluish basidiocarps, usually narrow allantoid and thin- to slightly thick-walled basidiospores (McGinty, 1909; Shen et al., 2019). In the current study, the phylogenetic analysis of *Cyanosporus* was carried out based on the combined sequence dataset of ITS + nLSU + nSSU + mtSSU + RPB1 + RPB2 + TEF rRNA gene regions. Combining morphological characters and molecular evidence, thirty-one species belonging to the *Postia caesia* complex are now recognized in *Cyanosporus*, including five new species and fifteen new combinations.

## Materials and Methods

### Taxa Sampling and Morphological Study

The examined specimens were deposited at the herbarium of the Institute of Microbiology, Beijing Forestry University (BJFC) with some duplicates at the Institute of Applied Ecology, Chinese Academy of Sciences (IFP). Morphological descriptions and abbreviations used in this study following Liu et al. (2019); Sun et al. (2020).

### DNA Extraction and Molecular Analyses

The procedures for DNA extraction and polymerase chain reaction (PCR) used in this study were the same as described by Chen et al. (2017); Song and Cui (2017). The primer pairs ITS5 and ITS4 for ITS regions, LR0R and LR7 for nLSU regions, NS1 and NS4 for nSSU regions, MS1 and MS2 for mtSSU regions, RPB1-Af and RPB1-Cr for RPB1 gene, fRPB2-f5F and bRPB2-7.1R for RPB2 gene, EF1-983 F and EF1-1567R for TEF gene used in this study are the same as previous studies (White et al., 1990; Rehner, 2001; Matheny et al., 2002; Matheny, 2005).

The PCR cycling schedules for different DNA sequences of ITS, nLSU, nSSU, mtSSU, RPB1, RPB2, and TEF genes used in this study followed those used in Liu et al. (2019); Shen et al. (2019), Zhu et al. (2019); Sun et al. (2020) with some modifications. The PCR products were purified and sequenced at the Beijing Genomics Institute (BGI), China, with the same primers. All newly generated sequences were deposited in GenBank (Table 1). Additional sequences for phylogenetic analyses were downloaded from GenBank (Table 1). All sequences were aligned in MAFFT 7 (Katoh and Standley, 2013^1^) and manually adjusted in BioEdit (Hall, 1999). Alignments were spliced in Mesquite (Maddison and Maddison, 2017). The missing sequences were coded as “N,” ambiguous nucleotides were coded as “N” following Chen et al. (2017). The final concatenated sequence alignment was deposited in TreeBase (^2^ submission ID: 27274).

**TABLE 1:** A list of species, specimens, and GenBank accession number of sequences used for phylogenetic analyses in this study.

Species	Sample no.	Locality	GenBank accessions						
			ITS	nLSU	nSSU	mtSSU	RPB1	RPB2	TEF
*Amaropostia stiptica*	Cui 10043	China	KX900906	KX900976	KX901119	KX901046	KX901167	KX901219	/
*A. stiptica*	Cui 10981	China	KX900907	KX900977	KX901120	KX901047	KX901168	KX901220	
*Amylocystis lapponica*	HHB-13400-Sp	United States	KC585237	KC585059	/	/	/	/	/
*A. lapponica*	OKM-4418-Sp	United States	KC585238	KC585060	/	/	/	/	/
*Antrodia serpens*	Dai 7465	Luxemburg	KR605813	KR605752	KR605913	KR606013	/	KR610832	KR610742
*A. serpens*	Dai 14850	Poland	MG787582	MG787624	MG787731	MG787674	/	MG787798	MG787849
*Cyanosporus alni*	Petr Vampola 12.10.1995	Slovakia	MG137026	/	/	/	/	/	/
*C. alni*	Cui 7185	China	KX900879	KX900949	KX901092	KX901017	KX901155	KX901202	KX901254
*C. alni*	Dai 14845	Poland	KX900880	KX900950	KX901093	KX901018	KX901156	KX901203	KX901255
C. arbuti	Viacheslav Spirin 8327	United States	MG137039	/	/	/	/	/	MG137132
*C. auricoma*	Dai 20992	Belarus	**MW182169**	/	/	/	/	/	/
*C. auricoma*	Cui 13518	China	KX900887	KX900957	KX901100	KX901025	/	KX901209	/
*C. auricoma*	Cui 13519	China	KX900888	KX900958	KX901101	KX901026	/	/	/
*C. auricoma*	Tuomo Niemelä 8310	Finland	MG137040	/	/	/	/	/	/
*C. auricoma*	Viacheslav Spirin 4586	Russia	MG137042	/	/	/	/	/	/
*C. bifaria*	Viacheslav Spirin 6402	Russia	MG137043	/	/	/	/	/	MG137133
*C. bifaria*	Cui 17445	China	**MW182170**	**MW182223**	**MW182187**	**MW182206**	/	**MW191562**	**MW191528**
*C. bifaria*	Cui 17806	China	**MW182171**	**MW182224**	**MW182188**	**MW182207**	**MW191546**	/	**MW191529**
*C. bubalinus*	Cui 16976	China	**MW182172**	**MW182225**	**MW182189**	**MW182208**	**MW191547**	**MW191563**	**MW191530**
*C. bubalinus*	Cui 16985	China	**MW182173**	**MW182226**	**MW182190**	**MW182209**	**MW191548**	**MW191564**	**MW191531**
*C. caesiosimulans*	Viacheslav Spirin 4199	Russia	MG137061	/	/	/	/	/	MG137140
*C. caesiosimulans*	Otto Miettinen 16976	United States	MG137054	/	/	/	/	/	MG137137
*C. caesius*	Otto Miettinen 14156	Finland	MG137048	/	/	/	/	/	MG137134
*C. caesius*	Gerhard Schuster 51	Germany	MG137045	/	/	/	/	/	/
*C. caesius* aff AR	CIEFAP 350	Argentina	JX090110	JX090130	/	/	/	/	/
*C. caesius* aff AR	CIEFAP 174	Argentina	JX090109	JX090129	/	/	/	/	/
*C. caesius* aff GB	K 32713	United Kingdom	AY599576	/	/	/	/	/	/
*C. caesius* aff GB	K 32425	United Kingdom	AY599575	/	/	/	/	/	/
*C. coeruleivirens*	Dai 11834	China	KF699119	/	/	/	/	/	/
*C. coeruleivirens*	Otto Miettinen 12214	Indonesia	MG137063	/	/	/	/	/	/
*C. coeruleivirens*	Dai 19220	China	**MW182174**	**MW182227**	**MW182191**	**MW182210**	**MW191549**	/	**MW191532**
*C. comata*	Otto Miettinen 14755,1	United States	MG137066	/	/	/	/	/	/
*C. comata*	Cui 18388	China	**MW182175**	**MW182228**	**MW182192**	/	**MW191550**	/	**MW191533**
*C. comata*	Cui 18546	China	/	**MW182229**	**MW182193**	/	/	/	**MW191534**
*C. cyanescens*	Otto Miettinen 13602	Finland	MG137067	/	/	/	/	/	MG137142
*C. cyanescens*	Otto Miettinen 15919,2	Spain	MG137071	/	/	/	/	/	MG137144
*C. fusiformis*	Cui 10775	China	KX900868	KX900938	KX901081	KX901006	/	KX901191	KX901245
*C. fusiformis*	Dai 15036	China	KX900867	KX900937	KX901080	KX901005	/	KX901190	KX901244
*C. glauca*	Viacheslav Spirin 5317	Russia	MG137078	/	/	/	/	/	/
*C. glauca*	Viacheslav Spirin 6580	Russia	MG137081	/	/	/	/	/	MG137145
*C. gossypina*	Bernard Rivoire 6658	France	/	/	/	/	/	/	MG137146
*C. hirsutus*	Cui 17050	China	**MW182176**	**MW182230**	**MW182194**	**MW182211**	**MW191551**	**MW191565**	**MW191535**
*C. hirsutus*	Cui 17053	China	**MW182177**	**MW182231**	**MW182195**	**MW182212**	**MW191552**	**MW191566**	**MW191536**
*C. hirsutus*	Cui 17055	China	**MW182178**	**MW182232**	**MW182196**	**MW182213**	**MW191553**	**MW191567**	**MW191537**
*C. hirsutus*	Cui 17083	China	**MW182179**	**MW182233**	**MW182197**	**MW182214**	**MW191554**	**MW191568**	**MW191538**
*C. livens*	Viacheslav Spirin 8728	United States	MG137090	/	/	/	/	/	MG137150
*C. livens*	Otto Miettinen 17177	United States	MG137082	/	/	/	/	/	MG137147
*C. luteocaesia*	Bernard Rivoire 2605	France	MG137091	/	/	/	/	/	/
*C. magna*	Cui 16983	China	**MW182180**	**MW182234**	**MW182198**	**MW182215**	**MW191555**	**MW191569**	**MW191539**
*C. magna*	Dai 10854	China	KF699117	/	/	/	/	/	/
*C. magna*	Otto Miettinen 10634	China	KC595944	KC595944	/	/	/	/	MG137151
*C. mediterraneocaesius*	LY BR 4274	France	KX900886	/	KX901099	KX901024	/	/	/
*C. microporus*	Cui 11014	China	KX900878	KX900948	KX901091	KX901016	/	KX901201	/
*C. microporus*	Dai 11717	China	KX900877	KX900947	KX901090	KX901015	/	KX901200	/
*C. nothofagicola*	Cui 16697	Australia	MW182181^a^	MW182235^a^	MW182199^a^	MW182216^a^	MW191556^a^	MW191570^a^	MW191540^a^
*C. nothofagicola*	Dai 18765	Australia	MW182182^a^	MW182236^a^	MW182200^a^	MW182217^a^	MW191557^a^	/	MW191541^a^
*C. piceicola*	Cui 10626	China	KX900862	KX900932	KX901075	KX901001	/	KX901185	/
*C. piceicola*	Cui 12158	China	KX900866	KX900936	KX901079	KX901004	KX901153	KX901189	KX901243
*C. populi*	Cui 17087a	China	**MW182183**	**MW182237**	**MW182201**	**MW182218**	**MW191558**	**MW191571**	**MW191542**
*C. populi*	Cui 17549	China	/	**MW182238**	**MW182202**	**MW182219**	**MW191559**	**MW191572**	**MW191543**
*C. populi*	Tuomo Niemelä 8379	Finland	MG137097	/	/	/	/	/	MG137154
*C. populi*	Otto Miettinen 17043	United States	MG137092	/	/	/	/	/	MG137153
*C. simulans*	Otto Miettinen 20422	Finland	MG137110	/	/	/	/	/	MG137160
*C. simulans*	Tuomo Niemelä 8846	Finland	MG137103	/	/	/	/	/	/
*C. subcaesius*	Josef Vlasák 0110/24	Czech Republic	MG137117	/	/	/	/	/	MG137164
*C. subcaesius*	Alix David 652	France	MG137116	/	/	/	/	/	/
*C. subhirsutus*	Cui 11330	China	KX900873	KX900943	KX901086	KX901011	/	KX901196	KX901250
*C. subhirsutus*	Dai 14892	China	KX900871	KX900941	KX901084	KX901009	/	KX901194	KX901248
*C. submicroporus*	Cui 16306	China	**MW182184**	**MW182239**	**MW182203**	**MW182220**	**MW191560**	**MW191573**	**MW191544**
*C. submicroporus*	Cui 17750	China	**MW182185**	**MW182240**	**MW182204**	**MW182221**	**MW191561**	/	**MW191545**
*C. submicroporus*	Cui 18156	China	**MW182186**	**MW182241**	**MW182205**	**MW182222**	/	**MW191574**	/
*C. subviridis*	Viacheslav Spirin 8774a	United States	MG137120	/	/	/	/	/	MG137166
*C. subviridis*	Reijo Penttilä 14376	Finland	/	/	/	/	/	/	MG137165
*C. tenuis*	Cui 10788	China	KX900885	KX900955	KX901098	KX901023	KX901161	KX901208	/
*C. tenuis*	Dai 12974	China	KX900884	KX900954	KX901097	KX901022	KX901160	KX901207	KX901258
*C. tricolor*	Cui 12233	China	KX900876	KX900946	KX901089	KX901014	/	KX901199	KX901253
*C. tricolor*	Cui 10790	China	KX900875	KX900945	KX901088	KX901013	/	KX901198	KX901252
*C. ungulatus*	Cui 10778	China	KX900870	KX900940	KX901083	KX901008	/	KX901193	KX901247
*C. ungulatus*	Dai 12897	China	KX900869	KX900939	KX901082	KX901007	KX901154	KX901192	KX901246
*C. yanae*	Heikki Kotiranta 27606	Russia	MG137122	/	/	/	/	/	MG137168
*C. yanae*	Heikki Kotiranta 27454	Russia	MG137121	/	/	/	/	/	MG137167
*Calcipostia guttulata*	Cui 10028	China	KF727433	KJ684979	KX901139	KX901066	KX901182	KX901237	KX901277
*C. guttulata*	KHL 11739 (GB)	Finland	EU118650	EU118650	/	/	/	/	/
*Cystidiopostia hibernica*	Cui 2658	China	KX900905	KX900975	KX901118	KX901045	/	KX901218	/
*C. hibernica*	K(M)17352	Austria	AJ006665	/	/	/	/	/	/
*Fuscopostia fragilis*	Cui 10020	China	KX900912	KX900982	KX901126	KX901054	/	KX901226	KX901270
*F. fragilis*	JV 0610-8	Czech	JF950573	/	/	/	/	/	/
*Oligoporus rennyi*	KEW 57	Unknown	AY218416	AF287876	/	/	/	/	/
*O. rennyi*	MR 10497	Argentina	JX090117	/	/	/	/	/	/
*Osteina obducta*	Cui 9959	China	KX900923	KX900993	KX901143	KX901070	/	KX901239	/
*O. obducta*	Cui 10074	China	KX900924	KX900994	KX901144	KX901071	/	KX901240	/
*Postia amurensis*	Cui 1044	China	KX900902	KX900972	/	KX901043	/	/	/
*P. amurensis*	Dai 903	China	KX900901	KX900971	/	KX901042	/	/	/
*P. hirsuta*	Cui 11180	China	KJ684971	KJ684985	/	KX901039	/	/	/
*P. hirsuta*	Cui 11237	China	KJ684970	KJ684984	KX901113	KX901038	/	/	KX901266
*P. lactea*	Cui 9319	China	KX900894	KX900964	KX901106	KX901031	KX901165	KX901213	KX901262
*P. lactea*	Cui 11511	China	KX900893	KX900963	KX901105	KX901030	KX901164	KX901212	KX901261
*Spongiporus leucospongia*	JV 0709/123-J	United States	KX900918	KX900988	KX901137	KX901064	/	/	KX901275
*S. leucospongia*	OKM 4335	United States	KC585395	KC585228	/	/	/	/	/

*New sequences for this study are indicated in bold.*

Phylogenetic analyses approaches used in this study followed Han et al. (2016); Cui et al. (2019). The congruences of the 7-gene (ITS, nLSU, nSSU, mtSSU, RPB1, RPB2, and TEF) were evaluated with the incongruence length difference (ILD) test (Farris et al., 1994) implemented in PAUP^∗^ 4.0b10 (Swofford, 2002), under heuristic search and 1000 homogeneity replicates. The sequences of *Antrodia serpens* (Fr.) Donk obtained from GenBank were used as outgroups for phylogenetic reconstruction. Maximum parsimony (MP) analysis was performed in PAUP^∗^ version 4.0b10 (Swofford, 2002). Maximum Likelihood (ML) analysis was performed in RAxmL v.7.2.8 with a GTR + G + I model (Stamatakis, 2006). Bayesian inference (BI) was calculated by MrBayes 3.1.2 (Ronquist and Huelsenbeck, 2003) with a general time reversible (GTR) model of DNA substitution and a gamma distribution rate variation across sites determined by MrModeltest 2.3 (Posada and Crandall, 1998; Nylander, 2004). Clade robustness was assessed using a bootstrap (BT) analysis with 1000 replicates (Felsenstein, 1985). Descriptive tree statistics tree length (TL), consistency index (CI), retention index (RI), rescaled consistency index (RC), and homoplasy index (HI) were calculated for each Most Parsimonious Tree (MPT) generated. The branch support was evaluated with a bootstrapping method of 1000 replicates (Hillis and Bull, 1993). Branches that received bootstrap supports for MP, ML greater than or equal to 75% and Bayesian posterior probabilities (BPP) greater than or equal to 0.95 were considered as significantly supported. The phylogenetic tree was visualized using FigTree v1.4.2^3^.

## Results

### Molecular Phylogeny

**FIGURE 1:**
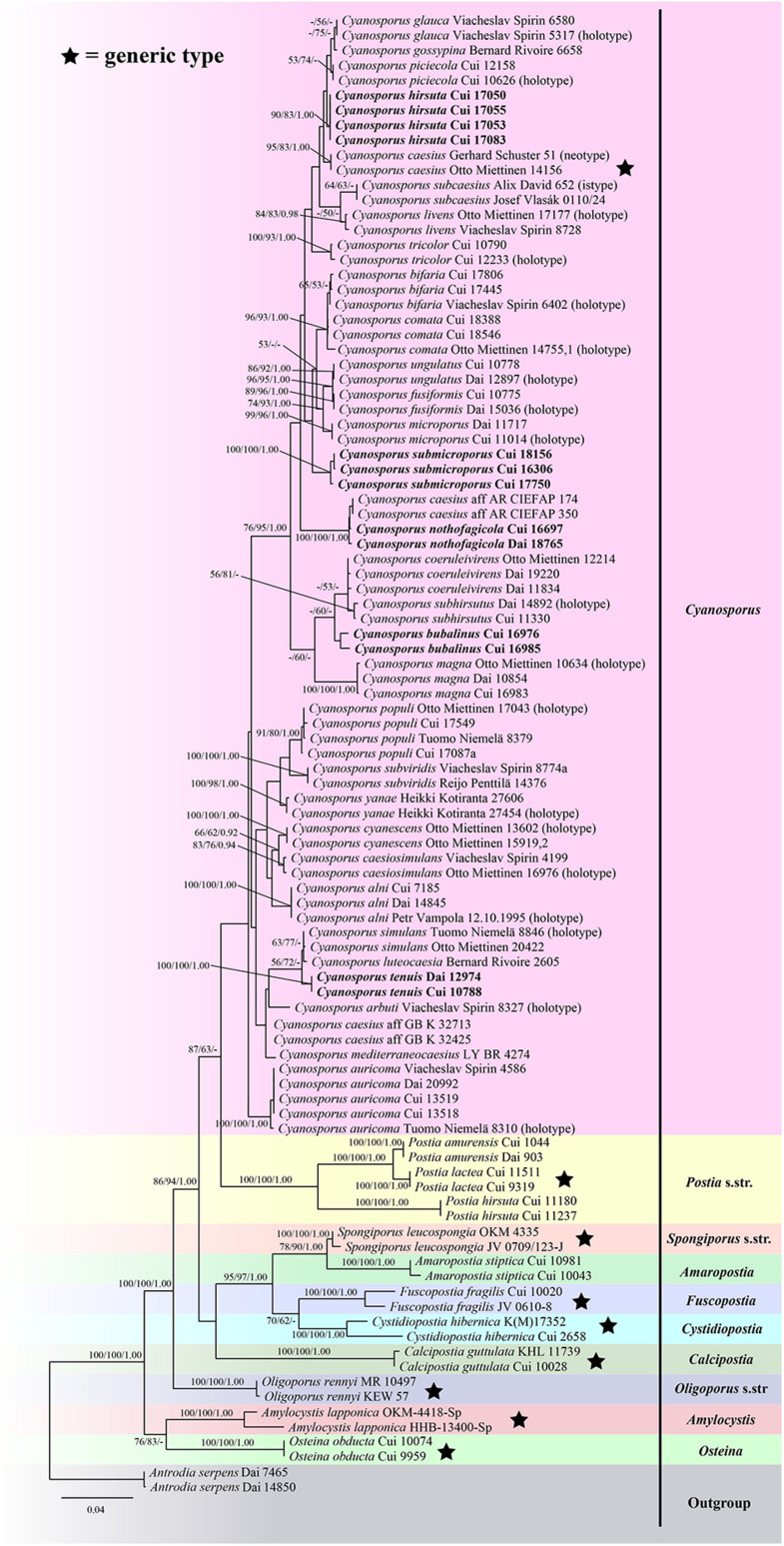
Maximum likelihood tree illustrating the phylogeny of *Cyanosporus* and its related genera in the antrodia clade based on sequences dataset of ITS + TEF. Branches are labeled with maximum likelihood bootstrap higher than 50%, parsimony bootstrap proportions higher than 50% and Bayesian posterior probabilities more than 0.90 respectively. Bold names = New species.

The ITS + TEF sequences dataset had an aligned length of 1166 characters, of which 646 characters were constant, 68 were variable and parsimony-uninformative, and 452 were parsimony-informative. MP analysis yielded 4 equally parsimonious trees (TL = 1983, CI = 0.417, RI = 0.726, RC = 0.303, HI = 0.583). The best model for the concatenate sequence dataset estimated and applied in the Bayesian inference was GTR + I + G with an equal frequency of nucleotides. ML analysis resulted in a similar topology as MP and Bayesian analyses, and only the ML topology is shown in Figure 1.

**FIGURE 2:**
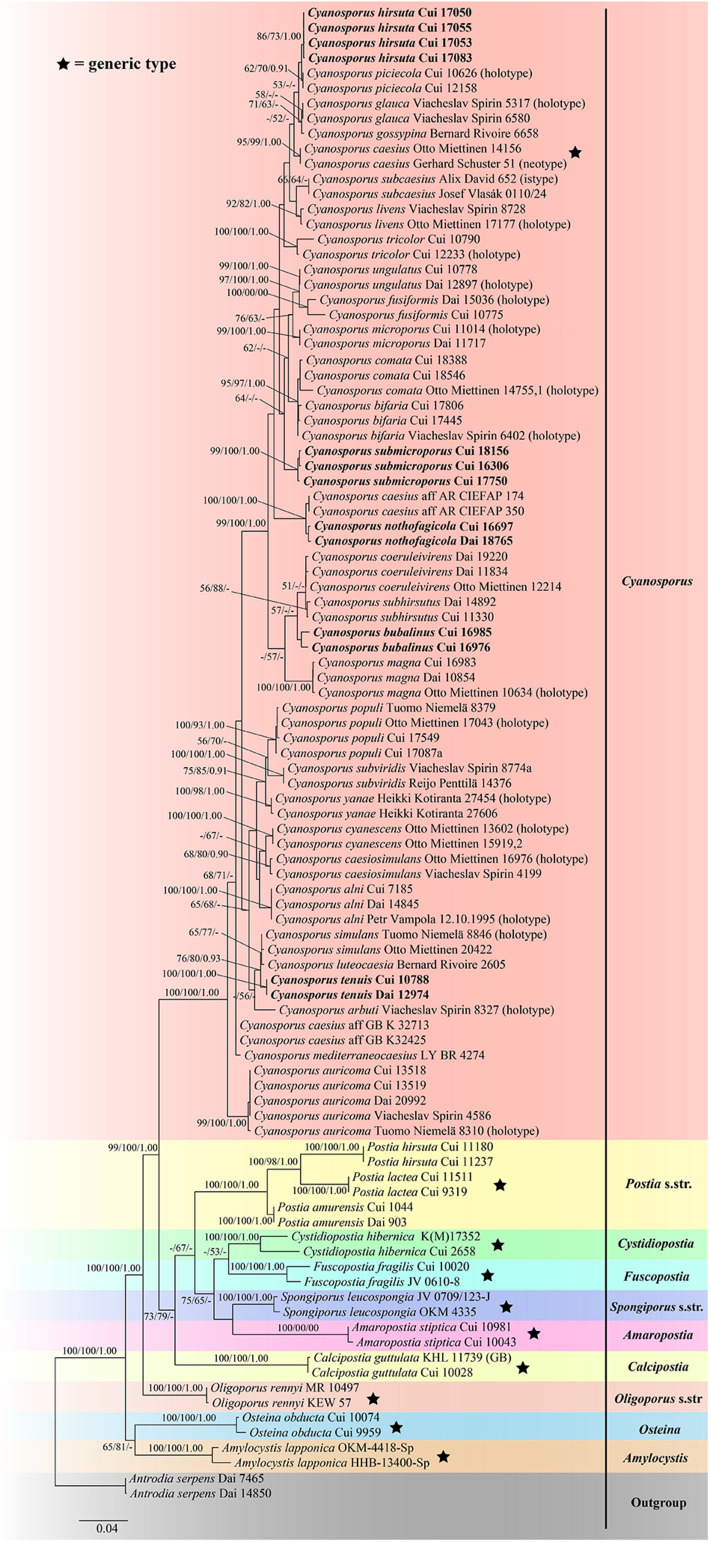
Maximum likelihood tree illustrating the phylogeny of *Cyanosporus* and its related genera in the antrodia clade based on the combined sequences dataset of ITS + nLSU + TEF. Branches are labeled with maximum likelihood bootstrap higher than 50%, parsimony bootstrap proportions higher than 50% and Bayesian posterior probabilities more than 0.90 respectively. Bold names = New species.

The combined three-gene (ITS + nLSU + TEF) sequences dataset had an aligned length of 2475 characters, of which 1752 characters were constant, 134 were variable and parsimony-uninformative, and 589 were parsimony-informative. MP analysis yielded 8 equally parsimonious trees (TL = 2222, CI = 0.473, RI = 0.770, RC = 0.365, HI = 0.527). The best model for the concatenate sequence dataset estimated and applied in the Bayesian inference was GTR + I + G with equal frequency of nucleotides. ML analysis resulted in a similar topology as MP and Bayesian analyses, and only the ML topology is shown in Figure 2.

**FIGURE 3:**
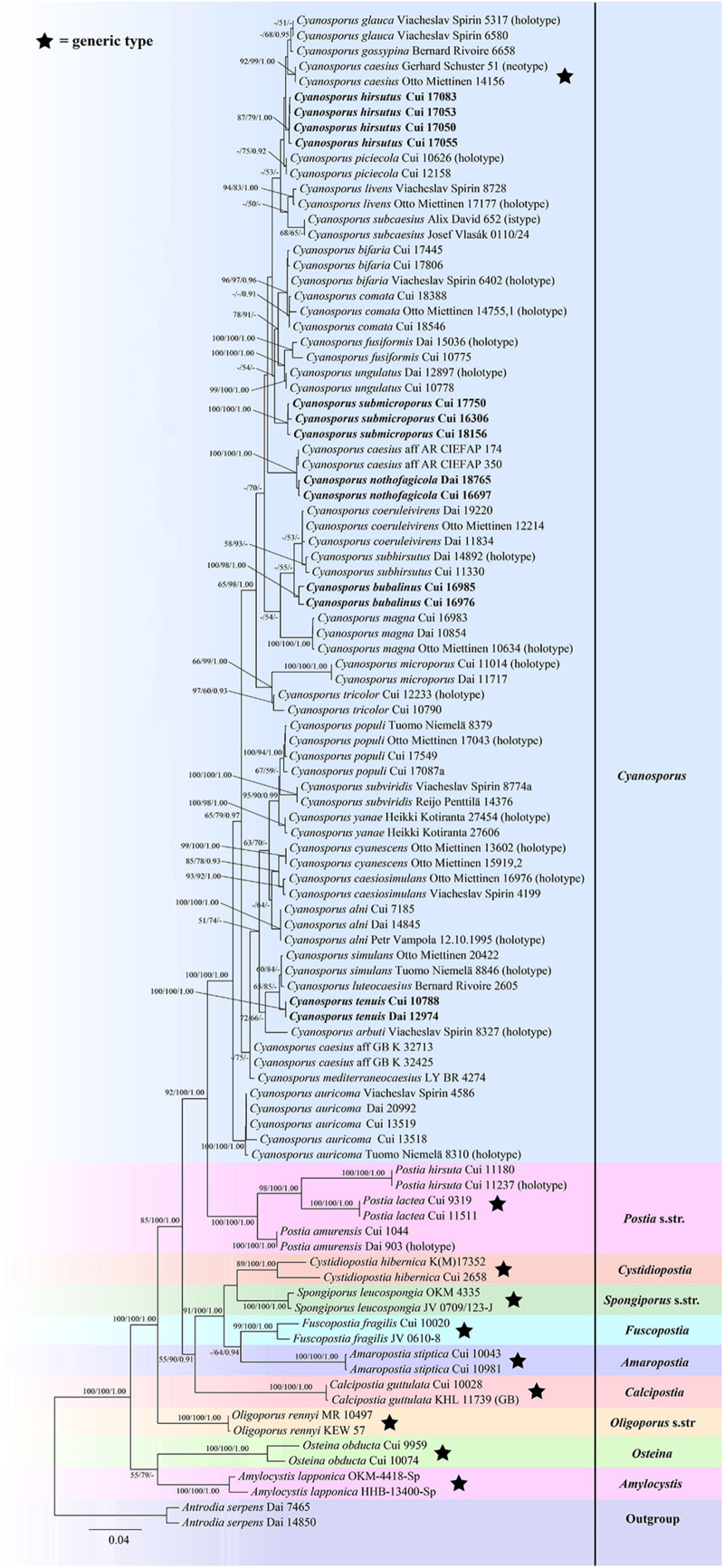
Maximum likelihood tree illustrating the phylogeny of *Cyanosporus* and its related genera in the antrodia clade based on the combined sequences dataset of ITS + nLSU + nSSU + mtSSU + RPB1 + RPB2 + TEF. Branches are labeled with maximum likelihood bootstrap higher than 50%, parsimony bootstrap proportions higher than 50% and Bayesian posterior probabilities more than 0.90 respectively. Bold names = New species.

The combined seven-gene (ITS + nLSU + nSSU + mtSSU + RPB1 + RPB2 + TEF) sequences dataset had an aligned length of 5855 characters, of which 4194 characters were constant, 300 were variable and parsimony-uninformative, and 1361 were parsimony-informative. MP analysis yielded 10 equally parsimonious trees (TL = 3951, CI = 0.585, RI = 0.806, RC = 0.471, HI = 0.415). The best model for the concatenate sequence dataset estimated and applied in the Bayesian inference was GTR + I + G with equal frequency of nucleotides. ML analysis resulted in a similar topology as MP and Bayesian analyses, and only the ML topology is shown in Figure 3.

The phylogenetic trees (Figures 1–3) inferred from ITS + TEF, ITS + nLSU + TEF and ITS + nLSU + nSSU + mtSSU + RPB1 + RPB2 + TEF gene sequences were obtained from 99 fungal samples representing 45 taxa of *Cyanosporus* and its related genera in the antrodia clade. Seventy-five samples representing thirty-one taxa of *Cyanosporus* clustered together and separated from species of *Postia* and other related genera.

### Taxonomy

**FIGURE 4:**
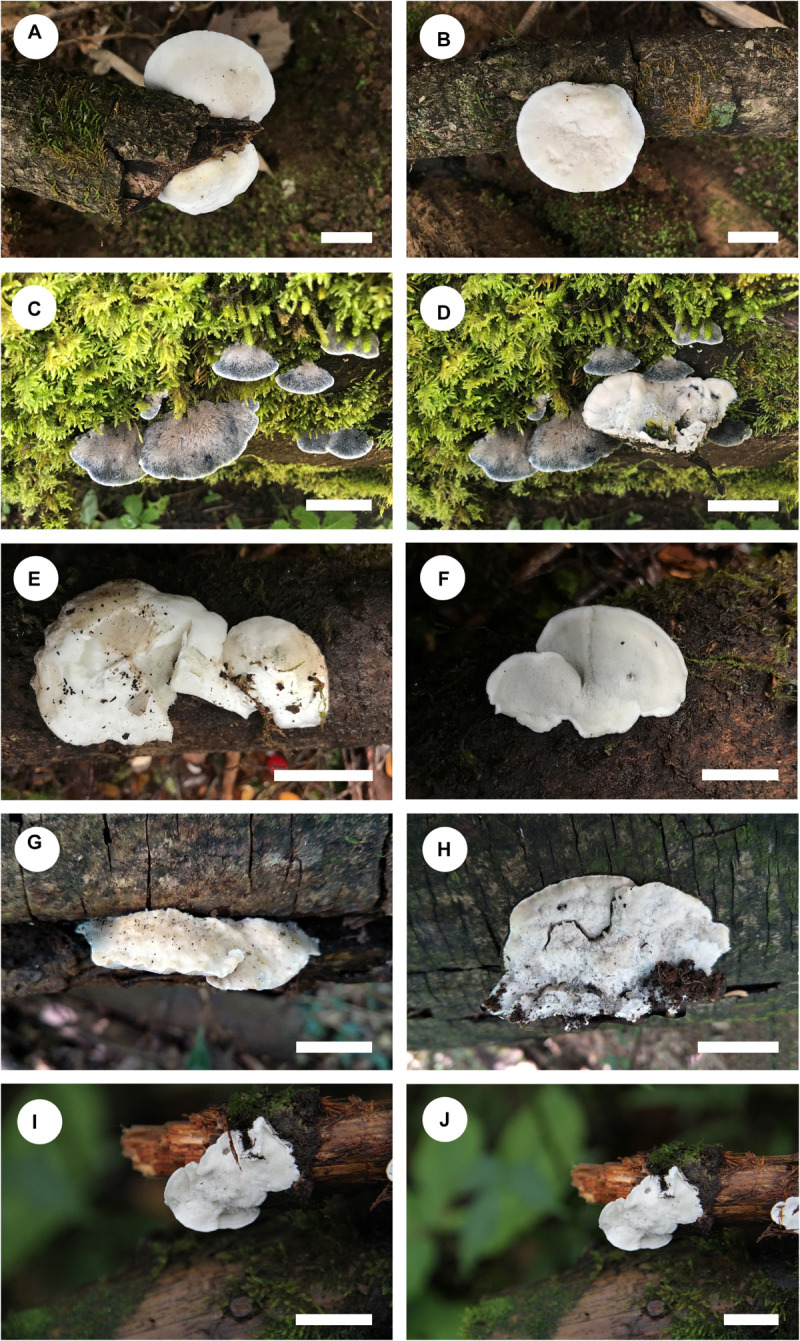
Basidiocarps of *Cyanosporus* species. **(A,B)** *C. bubalinus*; **(C,D)** *C. hirsutus*; **(E,F)** *C. nothofagicola*; **(G,H)**. *C. submicroporus*; **(I,J)**. *C. tenuis* (scale bars: **A,B,I,J** = 1 cm; **C,D,E,F,G,H** = 2 cm).

**FIGURE 5:**
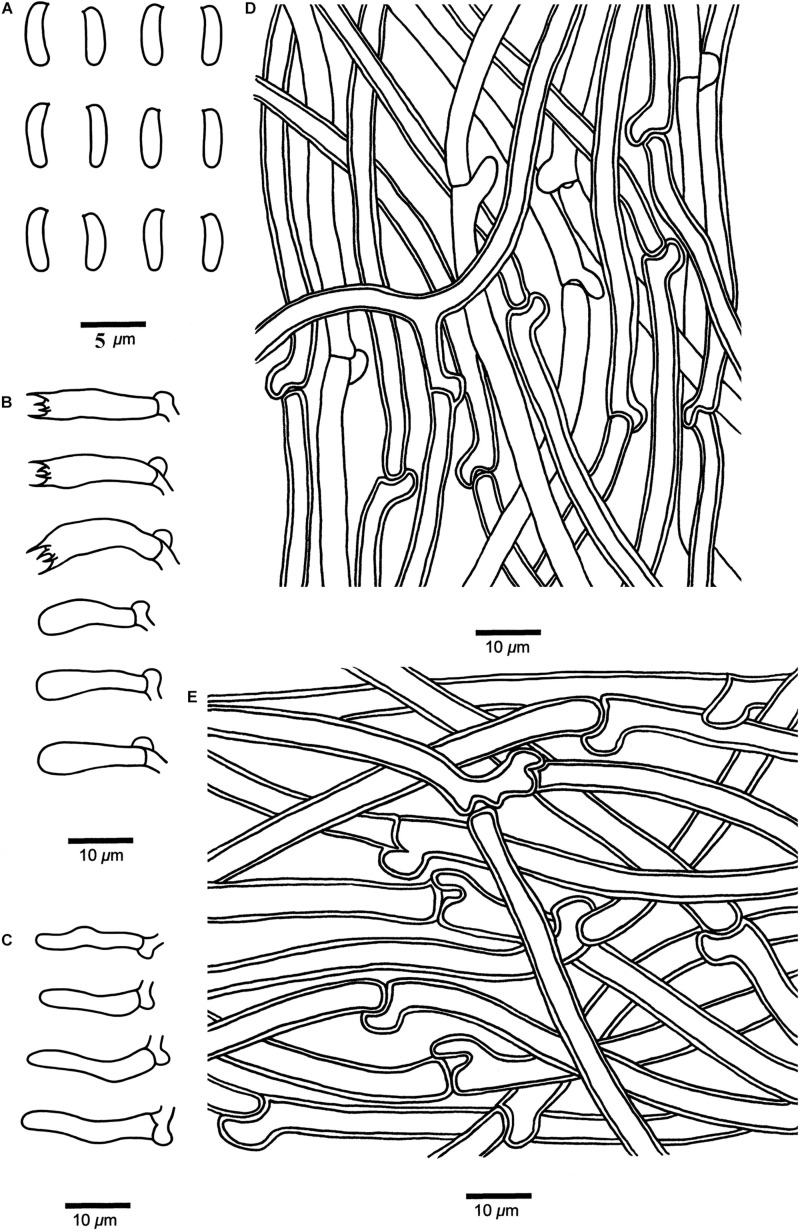
Microscopic structures of *Cyanosporus bubalinus* (drawn from the holotype). **(A)** Basidiospores; **(B)** Basidia and basidioles; **(C)** Cystidioles; **(D)** Hyphae from trama; **(E)** Hyphae from context. Bars: **(A)** = 5 μm; **(B–E)** = 10 μm.

***Cyanosporus bubalinus*** B.K. Cui & Shun Liu, **sp. nov.** (Figures 4A–B, 5)

MycoBank: MB 838417

Differs from other *Cyanosporus* species by its tomentose, cream to buff pileal surface when fresh becoming cream to pinkish buff when dry and a white to cream pore surface when fresh becoming straw yellow to buff when dry, and round to angular small pores (5–8 per mm).

*Type*. — **CHINA**. Yunnan Province, Binchuan County, Jizu Mountain, on fallen branch of *Pinus*, 14 September 2018, *Cui 16985* (holotype, BJFC).

*Etymology*. — *Bubalinus* (Lat.): refers to the cream to buff pileal surface.

*Basidiocarps*. — Annual, pileate, solitary, soft and watery when fresh, becoming soft corky to fragile upon drying. *Pileus* shell-shaped, projecting up to 2.5 cm, 3.5 cm wide and 1.5 cm thick at base. *Pileal surface* white to cream when fresh, finely tomentose, becoming cream to pinkish buff upon drying; margin acute. *Pore surface* white to cream when fresh, becoming straw yellow to buff when dry; sterile margin narrow to almost lacking; *pores* round to angular, 5–8 per mm; *dissepiments* thin, entire to lacerate. *Context* white, corky, up to 1.2 cm thick. *Tubes* cream, fragile, up to 5 mm long.

*Hyphal structure*. — Hyphal system monomitic; generative hyphae with clamp connections, IKI–, CB–; tissues unchanged in KOH.

*Context*. — Generative hyphae hyaline, slightly thick-walled with a wide lumen, occasionally branched, loosely interwoven, 2.5–7.3 μm in diam.

*Tubes*. — Generative hyphae hyaline, thin- to slightly thick-walled, occasionally branched, interwoven, 2–4.2 μm in diam. *Cystidia* absent; *cystidioles* present, fusoid, thin-walled, 13.3–23.4 × 2.9–4.2 μm. *Basidia* clavate, bearing four sterigmata and a basal clamp connection, 11.6–19.8 × 4.3–5.6 μm; *basidioles* dominant, in shape similar to basidia, but smaller.

*Basidiospores*. — Cylindrical, slightly curved, hyaline, thin- to slightly thick-walled, smooth, occasionally with small oily drops, IKI–, CB–, (4.2–)4.3–4.8 × 1.2–1.7(–1.8) μm, *L* = 4.65 μm, *W* = 1.55 μm, *Q* = 2.98–3.09 (*n* = 60/2).

*Notes*. — In the phylogenetic trees (Figures 1–3), two specimens of *Cyanosporus bubalinus* formed a highly supported lineage (Figures 1–3), closely related to *C. coeruleivirens* (Corner) B.K. Cui, Shun Liu & Y.C. Dai, *C. magna* (Miettinen) B.K. Cui & Shun Liu and *C. subhirsutus* B.K. Cui, L.L. Shen & Y.C. Dai. Morphologically, *C. coeruleivirens* differs by having a white, cream to bluish-grayish pore surface, smaller basidia (8.8–13.5 × 3.3–4.3 μm) and smaller basidiospores (3.8–4.8 × 1–1.3 μm) (Table 2); *C. magna* differs from *C. bubalinus* in its larger pores (4–5 per mm), smaller basidia (10–12.5 × 3.2–4 μm) and smaller basidiospores (3.6–4.4 × 1.0–1.2 μm) (Table 2); *C. subhirsutus* differs by having a hirsute pileal surface when dry, larger pores (2–3 per mm), smaller basidia (10–12 × 4–6 μm) and smaller basidiospores (4–4.5 × 0.9–1.3 μm) (Table 2). *Cyanosporus microporus* B.K. Cui, L.L. Shen & Y.C. Dai and *C. piceicola* B.K. Cui, L.L. Shen & Y.C. Dai were also discovered from Yunnan Province. *Cyanosporus microporus* differs from the new species by its narrower basidiospores (4.5–4.9 × 1–1.2 μm, Table 2); *C. piceicola* differs by having a velutinate, cream to clay-buff pileal surface, with bluish gray zonation when fresh, larger and round pores (3–5 per mm) and narrower basidiospores (4–4.5 × 0.9–1.3 μm). *Cyanosporus bifaria* (Spirin) B.K. Cui & Shun Liu, *C. glauca* (Spirin & Miettinen) B.K. Cui & Shun Liu and *C. subviridis* (Ryvarden & Guzmán) B.K. Cui & Shun Liu are similar to *C. bubalinus* as they all have pileate basidiocarps and similar pores, however, they differ by narrower tramal hyphae (2.5–3.8 μm in *C. bifaria*, 2.6–3.3 μm in *C. glauca*, 2.5–3.2 μm in *C. subviridis*) and smaller basidia (9.8–14.8 × 3.4–4.5 μm in *C. bifaria*, 9.8–14.8 × 3.1–4.3 μm in *C. glauca*, 10–13 × 3.2–4.4 μm in *C. subviridis*) (Table 2); in addition, they are distantly related from *C. bubalinus* in phylogeny.

**TABLE 2:** Comparisons of the main morphological characters of species in *Cyanosporus*.

Species	Distribution	Basidio-Carps	Pileal surface when dry	Pores per mm	Context hyphae (μm)	Tramal hyphae (μm)	Basidia (μm)	Basidiospores		Ref.
								L × W	Q = L/W	
								(μm)		
*C. alni*	China (Guizhou, Hebei), Czech Republic, Denmark, Finland, Germany, Norway, Poland, Russia, Slovakia	Pileate/Effused-reflexed	Velutinate	4–6	3.9–5.5	2.9–3.6	10–14.8 × 3.3–4.2	4.3–6.1 × 1.1–1.3	4.22	Miettinen et al., 2018
*C. arbuti*	United States	Pileate/Effused-reflexed	Glabrous	6–8	3.2–4.6	2.4–3.1	11–17 × 3.3–4.2	4.1–5.1 × 1–1.2	4	Miettinen et al., 2018
*C. auricoma*	China (Inner Mongolia), Finland, Poland, Russia	Pileate/Effused-reflexed/Resupinate	Hirsute	4–6	4.2–5.2	3.1–4.0	14–20 × 3.8–5.3	4.4–5.6 × 1.5–1.8	3.06	Miettinen et al., 2018
*C. bifaria*	China (Jilin, Sichuan), Japan, Russia	Pileate	Velutinate	6–8	4–7	2.5–3.8	9.8–14.8 × 3.4–4.5	3.7–4.4 × 1–1.2	3.6	Miettinen et al., 2018
*C. bubalinus*	China (Yunnan)	Pileate	Tomentose	5–8	2.5–7.3	2–4.2	11.6–19.8 × 4.3–5.6	4.3–4.8 × 1.5–2	2.6–2.83	this study
*C. caesius*	Czech Republic, Denmark, Finland, France, Germany, Russia, Slovakia, Spain, United Kingdom	Pileate/Effused-reflexed	Hirsute	4–5	3.7–5.2	2.8–3.6	10–15 × 3.7–4.5	4.1–5.3 × 1.3–1.7	3.13	Miettinen et al., 2018
*C. caesiosimulans*	Finland, Russia, United States	Pileate/Effused-reflexed	Glabrous	5–7	3.4–5.2	2.9–3.8	10.5–15.5 × 3.2–5.2	4.2–5.5 × 1.1–1.4	3.93	Miettinen et al., 2018
*C. coeruleivirens*	China (Hunan, Jilin, Zhejiang), Indonesia, Russia	Pileate	Velutinate	6–8	3.6–6	2.4–3.4	8.8–13.5 × 3.3–4.3	3.8–4.8 × 1–1.3	3.64	Miettinen et al., 2018
*C. comata*	China (Sichuan, Xizang), United States	Pileate/Effused-reflexed	Velutinate	4–6	4.2–5.3	2.8–3.8	8.8–14.2 × 3.7–4.9	4.1–4.9 × 1.1–1.3	3.62	Miettinen et al., 2018
*C. cyanescens*	Estonia, Finland, France, Poland, Russia, Spain, Sweden	Pileate/Effused-reflexed	Glabrous	5–6	4.1–5.2	2.9–3.7	11.4–19.8 × 3.7–5.4	4.7–6.1 × 1.1–1.6	3.92	Miettinen et al., 2018
*C. fusiformis*	China (Guizhou, Sichuan)	Pileate/Effused-reflexed	Tomentose	4–5	3–5	2–4	12–15 × 4.5–6	4.5–5.2 × 0.8–1.1	5.21–5.45	Shen et al., 2019
*C. glauca*	China (Jilin), Russia	Pileate	Hirsute	5–8	3.4–5.1	2.6–3.3	9.8–14.8 × 3.1–4.3	4.1–5.4 × 1.1–1.5	3.64	Miettinen et al., 2018
*C. gossypina*	France	Pileate	Glabrous	4–6	3.6–4.8	2.3–3	8.7–16.8 × 3.8–5	4.1–5.1 × 1.2–1.7	3.11	Miettinen et al., 2018
*C. hirsutus*	China (Yunnan)	Pileate	Hirsute	5–7	2.7–8.2	2–5	13.6–15.5 × 3.4–4.7	4–4.7 × 1.2–1.5	3.18–3.52	this study
*C. livens*	Canada, United States	Pileate	Velutinate	4–6	3.7–5.3	2.9–4	9.3–14.3 × 4–5.3	4.1–5.7 × 1.1–1.5	3.74	Miettinen et al., 2018
*C. luteocaesius*	France	Pileate/Effused-reflexed	Tomentose	3–5	4.1–5.8	2.7–3.2	11.1–16.2 × 4–5.2	4.3–6.1 × 1.5–1.9	3.02	Miettinen et al., 2018
*C. magna*	China (Jilin, Hainan, Yunnan)	Pileate	Velutinate	4–5	4.2–6	2.2–3.3	10–12.5 × 3.2–4	3.6–4.4 × 1–1.2	3.51	Miettinen et al., 2018
*C. mediterraneocaesius*	France, Spain	Pileate/Effused-reflexed	Velutinate	5–6	3.1–4	2.3–3.2	12–18.5 × 3.5–4.6	4.2–5.8 × 1.3–1.7	3.26	Miettinen et al., 2018
*C. microporus*	China (Yunnan)	Pileate	Velutinate	6–8	3.5–6	2–4	11–13.5 × 4–5	4.5–4.9 × 1–1.2	4.47–4.52	Shen et al., 2019
*C. nothofagicola*	Australia	Pileate/Effused-reflexed	Tomentose	4–6	2.3–7.5	2–5	13.6–17.2 × 3.4–4.2	3.8–5 × 1–1.7	4.08–4.22	this study
*C. piceicola*	China (Sichuan, Xizang, Yunnan)	Pileate	Velutinate	3–5	5–7	2.5–4	13–16 × 4–5	4–4.5 × 0.9–1.3	3.75–3.97	Shen et al., 2019
*C. populi*	China (Jilin, Sichuan, Yunnan), Finland, Norway, Poland, Russia, United States	Pileate/Effused-reflexed	Glabrous	5–7	3.2–4.8	2.7–3.3	10–16 × 3.5–4.2	4.2–5.6 × 1–1.3	4.14	Miettinen et al., 2018
*C. simulans*	Canada, China, Estonia, Finland, France, Germany, Norway, Russia, United States	Pileate/Effused-reflexed/Resupinate	Glabrous	5–7	3.9–5	2.8–3.6	10–14.8 × 3.7–5.2	4.4–6.3 × 1.3–1.8	3.6	Miettinen et al., 2018
*C. subcaesius*	Czech Republic, Finland, France, Russia, United Kingdom	Pileate/Effused-reflexed	Glabrous	4–6	4.2–6.6	3.1–4.1	10.3–17.8 × 3.3–4.6	4–5.3 × 1–1.4	3.8	Miettinen et al., 2018
*C. subhirsutus*	China (Guizhou, Fujian, Yunnan)	Pileate	Hirsute	2–3	4–6	3–4.5	10–12 × 4–6	4–4.5 × 0.9–1.3	3.67–3.79	Shen et al., 2019
*C. submicroporus*	China (Sichuan, Yunnan)	Pileate	Velutinate	6–9	2.3–6.2	2–4.8	12.2–20.5 × 3.4–5.6	3.6–4.7 × 1–1.3	3.45–3.52	this study
*C. subviridis*	Finland, Mexico, United States	Pileate	Glabrous	6–8	4.8–5.8	2.5–3.2	10–13 × 3.2–4.4	3.8–4.5 × 1–1.3	3.58	Miettinen et al., 2018
*C. tricolor*	China (Sichuan, Xizang)	Pileate	Velutinate	4–5	3–5	2–3	12–15 × 4–5	4–4.8 × 0.8–1.2	4.55–4.87	Shen et al., 2019
*C. tenuis*	China (Sichuan)	Pileate/Effused-reflexed	Tomentose	5–7	2.6–7	2.2–4.8	18.2–27.6 × 3.7–6	4.7–6 × 1.3–2	2.89–2.93	This study
*C. ungulatus*	China (Sichuan)	Pileate	Glabrous	4–6	2.5–4.5	2–3	12–15 × 4–5	4.5–5 × 0.9–1.2	4.79–4.83	Shen et al., 2019
*C. yanae*	Russia	Pileate/Effused-reflexed	Glabrous	5–7	3–4	2.2–2.9	9–14 × 3.5–4.2	4.3–5.8 × 1.2–1.6	3.56	Miettinen et al., 2018

Additional specimen (paratype) examined: **CHINA**. Yunnan Province, Binchuan County, Jizu Mountain, on wood of *Pinus*, 14 September 2018, *Cui 16976* (BJFC).

**FIGURE 6:**
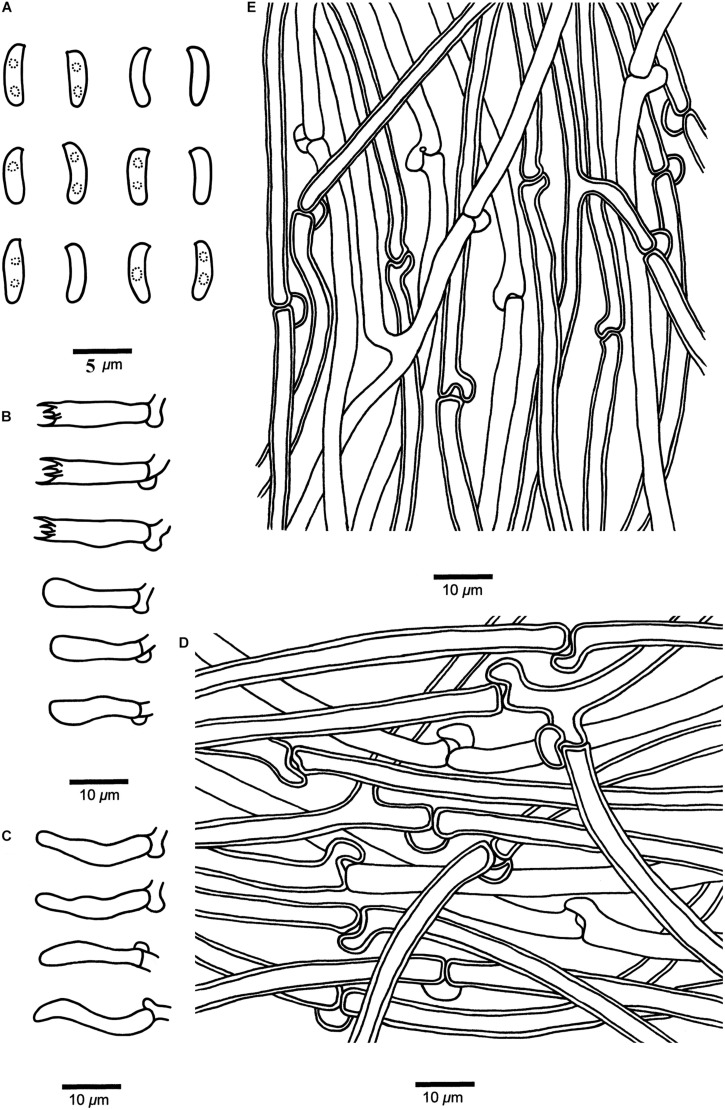
Microscopic structures of *Cyanosporus hirsutus* (drawn from the holotype). **(A)** Basidiospores; **(B)** Basidia and basidioles; **(C)** Cystidioles; **(D)** Hyphae from trama; **(E)** Hyphae from context. Bars: **(A)** = 5 μm; **(B–E)** = 10 μm.

***Cyanosporus hirsutus*** B.K. Cui & Shun Liu, **sp. nov.** (Figures 4C–D, 6)

MycoBank: MB 838418

Differs from other *Cyanosporus* species by its flabelliform to semicircular and distinctly hirsute pileus with ash gray to light grayish brown pileal surface, and cylindrical and slightly curved basidiospores (4.1–4.7 × 1.2–1.5 μm).

*Type*. — **CHINA**. Yunnan Province, Lijiang, Yulong Snow Mountain, on fallen trunk of *Picea*, 14 September 2018, *Cui 17083* (holotype, BJFC).

*Etymology*. — *Hirsutus* (Lat.): refers to the distinctly hirsute pileal surface.

*Basidiocarps*. — Annual, pileate, soft corky, without odor or taste when fresh, becoming corky to fragile upon drying. *Pileus* flabelliform to semicircular, projecting up to 5.2 cm, 9.5 cm wide and 1.5 cm thick at base. *Pileal surface* ash gray to light grayish brown with bluish gray zone when fresh, becoming grayish to grayish brown when dry, distinctly hirsute; margin acute. *Pore surface* cream when fresh, becoming straw yellow to olivaceous buff when dry; sterile margin narrow to almost lacking; *pores* angular, 5–7 per mm; *dissepiments* thin, entire. *Context* white, soft corky, up to 9 mm thick. *Tubes* cream, fragile, up to 7 mm long.

*Hyphal structure*. — Hyphal system monomitic; generative hyphae with clamp connections, IKI–, CB–; tissues unchanged in KOH.

*Context*. — Generative hyphae hyaline, thin- to slightly thick-walled with a wide lumen, occasionally branched, loosely interwoven, 2.7–8.2 μm in diam.

*Tubes*. — Generative hyphae hyaline, thin- to slightly thick-walled with a wide lumen, occasionally branched, interwoven, 2–5 μm in diam. *Cystidia* absent; *cystidioles* present, fusoid, thin-walled, 13.2–22.5 × 2.7–4.3 μm. *Basidia* clavate, bearing four sterigmata and a basal clamp connection, 13.6–15.5 × 3.4–4.7 μm; *basidioles* dominant, in a shape similar to basidia, but smaller.

*Basidiospores.* — Cylindrical, slightly curved, hyaline, thin- to slightly thick-walled, smooth, occasionally with small oily drops, IKI–, CB–, 4–4.7(–4.9) × (1–)1.2–1.5(–1.8) μm, *L* = 4.42 μm, *W* = 1.33 μm, *Q* = 3.18–3.52 (*n* = 90/3).

*Notes*. — Phylogenetically, *Cyanosporus hirsutus* grouped together with *C. caesius*, *C. glauca*, *C. gossypina* (Moug. & Lév.) B.K. Cui & Shun Liu, *C. mediterraneocaesius* (M. Pieri & B. Rivoire) B.K. Cui, L.L. Shen & Y.C. Dai and *C. piceicola*. Morphologically, *C. hirsutus* is similar to *C. caesius* as both species share hairy and distinctly bluish basidiocarps, but *C. caesius* differs from *C. hirsutus* by its larger pores (4–5 per mm), and narrower hyphae of the context (3.7–5.2) (Table 2); *C. hirsutus* and *C. glauca* have similar-sized basidiospores, but *C. glauca* has a plumbeous to bluish gray or grayish brown pileal surface (Miettinen et al., 2018); *C. hirsutus* and *C. gossypina* have similar-sized pores and basidiospores, but *C. gossypina* has smaller basidiocarps with a cream to light gray pileal surface (Miettinen et al., 2018); *C. hirsutus* and *C. mediterraneocaesius* share similar-sized and similar-shaped pores, but *C. mediterraneocaesius* has larger basidiospores (4.2–5.8 × 1.3–1.7) (Table 2); *C. hirsutus* and *C. piceicola* both have a flabelliform pileus, with bluish gray zonate pileal surface, similar-sized pores and basidiospores, but *C. piceicola* has smaller basidiocarps (projecting up to 3 cm, 5.5 cm wide and 1.8 cm thick at base), thinner hyphae in the context (5–7 μm) and smaller basidia (13–16 × 4–5 μm) (Shen et al., 2019). *Cyanosporus microporus* and *C. subhirsutus* both have pileate basidiocarps with a blue tint to the pileal surface and slightly thick-walled basidiospores like *C. hirsutus*, but *C. microporus* differs in its smaller pores (6–8 per mm) and basidia (11–13.5 × 4–5 μm) (Table 2); *C. subhirsutus* differs in having larger pores (2–3 per mm) and smaller basidia (10–12 × 4–6 μm) (Table 2).

Additional specimens (paratypes) examined. **CHINA**. Yunnan Province, Lijiang, Yulong Snow Mountain, on fallen trunk of *Picea*, 14 September 2018, *Cui 17050*, *17053*, *17055*, *17070*, *17082* (BJFC).

**FIGURE 7:**
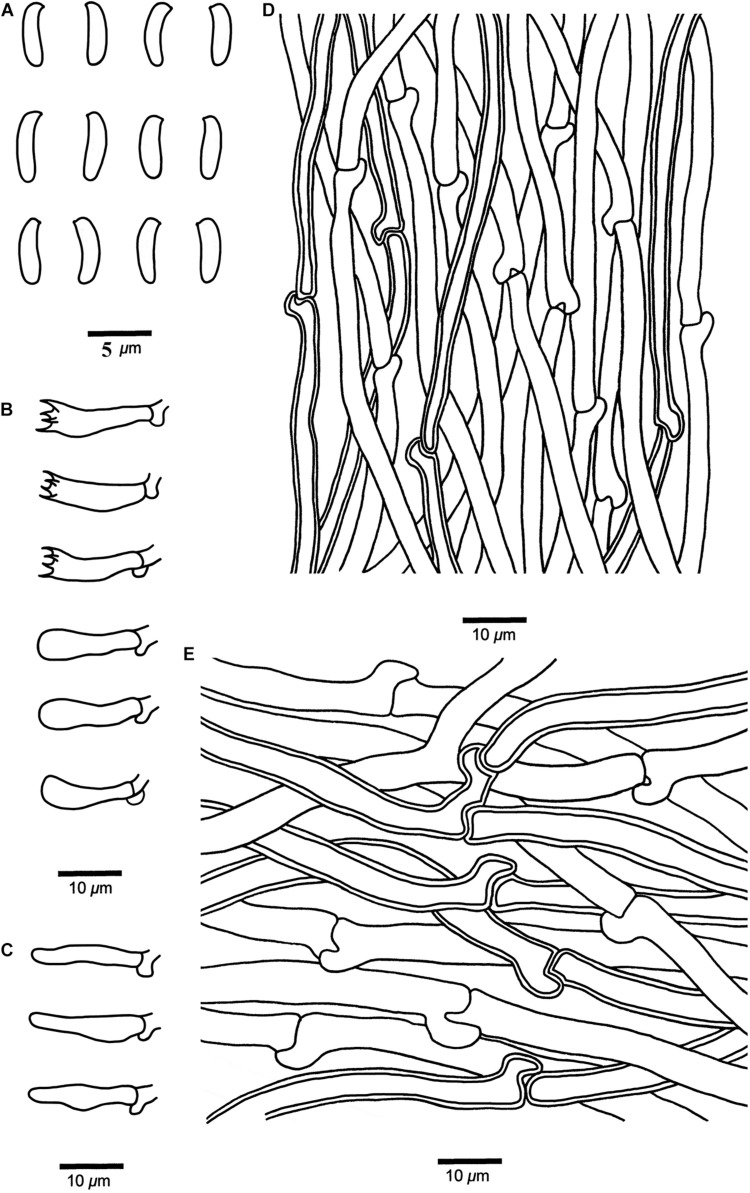
Microscopic structures of *Cyanosporus nothofagicola* (drawn from the holotype). **(A)** Basidiospores; **(B)** Basidia and basidioles; **(C)** Cystidioles; **(D)** Hyphae from trama; **(E)** Hyphae from context. Bars: **(A)** = 5 μm; **(B–E)** = 10 μm.

***Cyanosporus nothofagicola*** B.K. Cui, Shun Liu & Y.C. Dai, **sp. nov.** (Figures 4E–F, 7)

MycoBank: MB 838419

Differs from other *Cyanosporus* species by its flabelliform to semicircular pileus with pale mouse gray to buff yellow pileal surface and cream to buff yellow pore surface when dry, cylindrical to allantoid basidiospores (3.8–5 × 1–1.7 μm), and growth on *Nothofagus*.

*Type*. — **AUSTRALIA**. Tasmania, Arve River Streamside Reserve, on fallen branch of *Nothofagus*, 15 May 2018, *Cui 16697* (holotype, BJFC).

*Etymology*. — (Lat.): refers to the frequent occurrence on *Nothofagus*.

*Basidiocarps*. — Annual, effused-reflexed to pileate, solitary, soft and watery, without odor or taste when fresh, becoming soft corky to fragile upon drying. *Pileus* flabelliform to semicircular, projecting up to 2.4 cm, 4.2 cm wide and 0.7 cm thick at base. *Pileal surface* buff to olivaceous buff when fresh, finely tomentose, becoming smooth, pale mouse gray to buff yellow when dry; margin acute. *Pore surface* white to cream when fresh, becoming cream to buff yellow when dry; sterile margin narrow to almost lacking; *pores* angular, 4–6 per mm; *dissepiments* thin, entire to lacerate. *Context* white, soft corky, up to 4 mm thick. *Tubes* cream, fragile, up to 3 mm long.

*Hyphal structure*. — Hyphal system monomitic; generative hyphae with clamp connections, IKI–, CB–; tissues unchanged in KOH.

*Context*. — Generative hyphae hyaline, thin- to slightly thick-walled with a wide lumen, occasionally branched, loosely interwoven, 2.3–7.5 μm in diam.

*Tubes*. — Generative hyphae hyaline, thin- to slightly thick-walled with a wide lumen, occasionally branched, interwoven, 2–5 μm in diam. *Cystidia* absent; *cystidioles* present, fusoid, thin-walled, 14.2–16.7 × 2.6–5.2 μm. *Basidia* clavate, bearing four sterigmata and a basal clamp connection, 13.6–17.2 × 3.4–4.2 μm; *basidioles* dominant, in shape similar to basidia, but smaller.

*Basidiospores.* — Cylindrical to allantoid, slightly curved, hyaline, thin- to slightly thick-walled, smooth, IKI–, CB–, 3.8–5 × (0.8–)1–1.7 μm, *L* = 4.62 μm, *W* = 1.15 μm, *Q* = 4.08–4.22 (*n* = 60/2).

*Notes*. — *Cyanosporus caesius* is also found in Tasmania, Australia. It has pileate or effused reflexed basidiocarps with a tomentose pileal surface and similar pores to *C. nothofagicola*, but it differs from the later by its plumbeous to bluish gray or grayish brown pileal surface (Miettinen et al., 2018).

Additional specimen (paratype) examined. **AUSTRALIA**. Tasmania, Arve River Streamside Reserve, on fallen trunk of *Nothofagus*, 15 May 2018, *Dai 18765* (BJFC).

**FIGURE 8:**
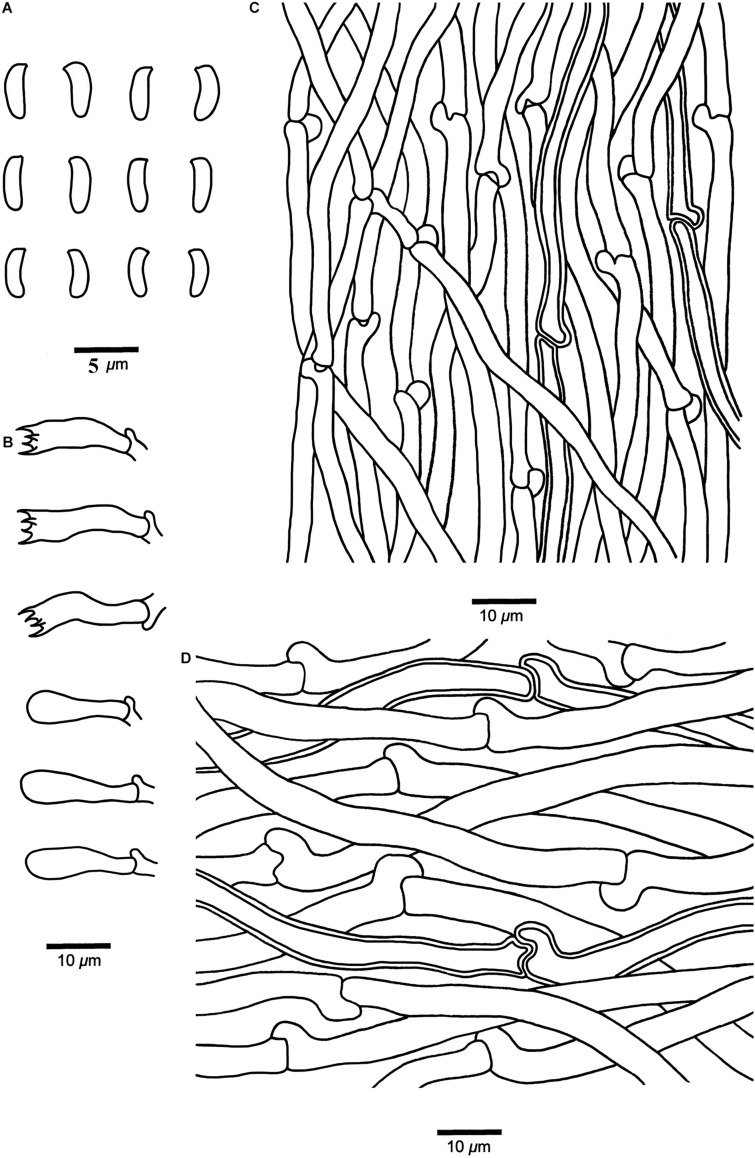
Microscopic structures of *Cyanosporus submicroporus* (drawn from the holotype). **(A)** Basidiospores; **(B)** Basidia and basidioles; **(C)**. Hyphae from trama; **(D)** Hyphae from context. Bars: **(A)** = 5 μm; **(B–D)** = 10 μm.

***Cyanosporus submicroporus*** B.K. Cui & Shun Liu, **sp. nov.** (Figures 4G–H, 8)

MycoBank: MB 838420

Differs from other *Cyanosporus* species by its cream to pinkish buff pileal surface and white to smoke gray pore surface when fresh, buff to buff yellow pileal surface and buff to olivaceous buff pore surface when dry.

*Type*. — **CHINA**. Yunnan Province, Baoshan, Gaoligongshan Nature Reserve, on angiosperm wood, 8 November 2019, *Cui 18156* (holotype, BJFC).

*Etymology*. — *Submicroporus* (Lat.): refers to the new species resembling *Cyanosporus microporus* in morphology.

*Basidiocarps*. — Annual, pileate, soft and watery, without odor or taste when fresh, becoming corky to woody hard upon drying. *Pileus* flabelliform to semicircular, projecting up to 3.2 cm, 6.5 cm wide and 1.3 cm thick at base. *Pileal surface* cream to pinkish buff when fresh, velutinate, becoming rugose, buff to buff yellow when dry; margin acute. *Pore surface* white to smoke gray when fresh, becoming buff to olivaceous buff when dry; sterile margin narrow to almost lacking; *pores* round, 6–9 per mm; *dissepiments* thin, entire. *Context* cream to buff, corky, up to 5 mm thick. *Tubes* pale mouse gray to cream, fragile, up to 7 mm long.

*Hyphal structure*. — Hyphal system monomitic; generative hyphae with clamp connections, IKI–, CB–; tissues unchanged in KOH.

*Context*. — Generative hyphae hyaline, thin- to slightly thick-walled with a wide lumen, rarely branched, loosely interwoven, 2.3–6.2 μm in diam.

*Tubes*. — Generative hyphae hyaline, thin- to slightly thick-walled with a wide lumen, occasionally branched, interwoven, 2–4.8 μm in diam. *Cystidia* and *cystidioles* absent. *Basidia* clavate, bearing four sterigmata and a basal clamp connection, 12.2–20.5 × 3.4–5.6 μm; *basidioles* dominant, in shape similar to basidia, but smaller.

Basidiospores. — Allantoid, slightly curved, hyaline, thin- to slightly thick-walled, smooth, occasionally with small oil drops, IKI–, weakly CB+, 3.6–4.7 × (0.9–)1–1.3 μm, *L* = 4.18 μm, *W* = 1.19 μm, *Q* = 3.45–3.52 (*n* = 90/3).

*Notes*. — In the phylogenetic tree the three specimens of *Cyanosporus submicroporus* formed a highly supported lineage (Figures 1-3), and are usually grouped together with *C. bifaria*, *C. comata* (Miettinen) B.K. Cui & Shun Liu, *C. fusiformis* B.K. Cui, L.L. Shen & Y.C. Dai and *C. ungulatus* B.K. Cui, L.L. Shen & Y.C. Dai. Morphologically, both *C. submicroporus* and *C. bifaria* have similar pores and basidiospores, but *C. bifaria* differs in having ochraceous hues, a strigose pileal surface, and smaller basidia (9.8–14.8 × 3.4–4.5 μm) (Table 2); *C. comata* has similar basidiospores to *C. submicroporus*, but differs in having conchate or effused-reflexed basidiocarps, and smaller basidia (8.8–14.2 × 3.7–4.9 μm) (Table 2); *C. fusiformis* and *C. ungulatus* differ in having larger pores (Table 2). *Cyanosporus submicroporus* is similar to *C. microporus* by having pileate basidiocarps with a velutinate pileal surface, and small and angular pores, but *C. microporus* differs in having thick-walled tramal generative hyphae, smaller basidia (11–13.5 × 4–5 μm) and longer basidiospores (4.5–4.9 × 1–1.2 μm) (Table 2).

Additional specimens (paratypes) examined. **CHINA**. Sichuan Province, Shimian County, on fallen angiosperm trunk, 14 September 2019, *Cui 17750* (BJFC); Yunnan Province, Chuxiong, Zixishan Nature Reserve, on fallen angiosperm trunk, 20 September 2017, *Cui 16306* (BJFC).

**FIGURE 9:**
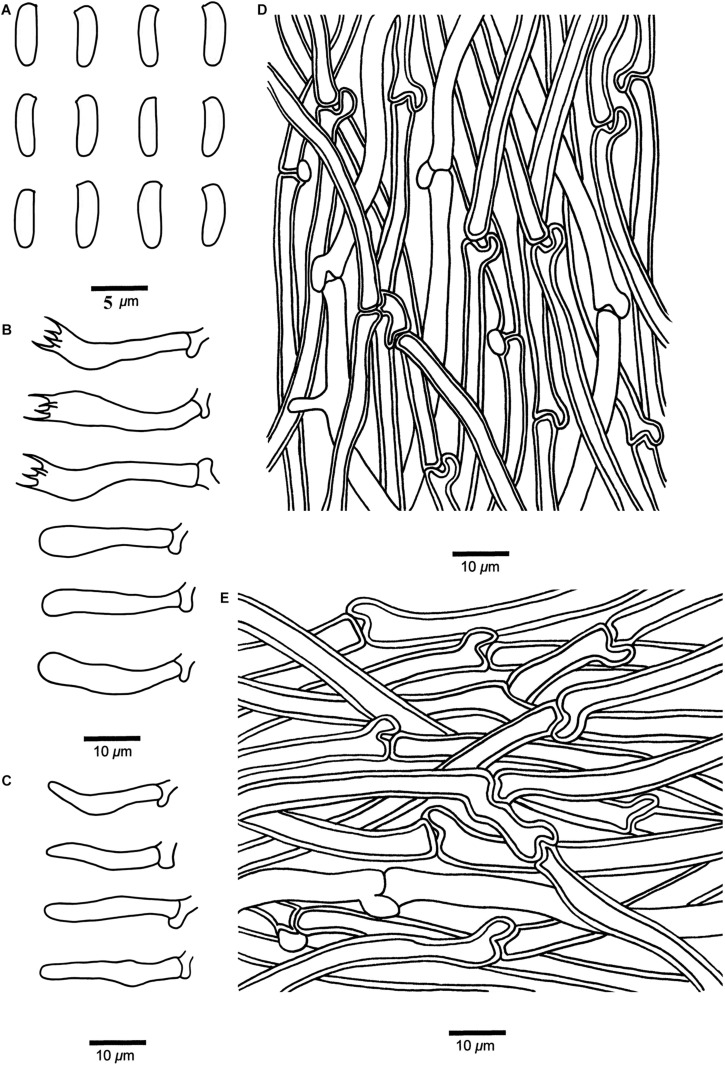
Microscopic structures of *Cyanosporus tenuis* (drawn from the holotype). **(A)** Basidiospores; **(B)** Basidia and basidioles; **(C)** Cystidioles; **(D)** Hyphae from trama; **(E)** Hyphae from context. Bars: **(A)** = 5 μm; **(B–E)** = 10 μm.

***Cyanosporus tenuis*** B.K. Cui, Shun Liu & Y.C. Dai, **sp. nov.** (Figures 4I–J, 9)

MycoBank: MB 838421

Differs from other *Cyanosporus* species by the thin basidiocarps, larger cystidioles (16.4–25.4 × 2.8–4.2 μm), and bigger basidiospores (4.7–6 × 1.3–2 μm).

*Type*. — **CHINA**. Sichuan Province, Luding County, Hailuogou Forest Park, on fallen trunk of *Picea*, 20 October 2012, *Cui 10788* (BJFC).

*Etymology*. — *Tenuis* (Lat.): refers to the thin basidiocarps.

*Basidiocarps*. — Annual, effused-reflexed to pileate, soft corky, without odor or taste when fresh, becoming corky to fragile upon drying. *Pileus* flabelliform, projecting up to 1.5 cm, 1.8 cm wide and 0.4 cm thick at base. *Pileal surface* buff to olivaceous buff when fresh, finely tomentose, becoming cream to olivaceous buff when dry; margin acute. *Pore surface* white to cream when fresh, becoming buff yellow to pinkish buff when dry; sterile margin narrow to almost lacking; pores angular, 5–7 per mm; dissepiments thin, entire to lacerate. *Context* cream, soft corky, up to 2 mm thick. *Tubes* pinkish buff, fragile, up to 2 mm long.

*Hyphal structure*. — Hyphal system monomitic; generative hyphae with clamp connections, IKI–, CB–; tissues unchanged in KOH.

*Context*. — Generative hyphae hyaline, thin- to slightly thick-walled with a wide lumen, occasionally branched, loosely interwoven, 2.6–7 μm in diam.

*Tubes*. — Generative hyphae hyaline, thin- to slightly thick-walled with a wide lumen, occasionally branched, interwoven, 2.2–4.8 μm in diam. *Cystidia* absent; *cystidioles* present, fusoid, thin-walled, 16.4–25.4 × 2.8–4.2 μm. *Basidia* clavate, bearing four sterigmata and a basal clamp connection, 18.2–27.6 × 3.7–6 μm; *basidioles* dominant, in a shape similar to basidia, but smaller.

*Basidiospores.* — Cylindrical, slightly curved, hyaline, thin- to slightly thick-walled, smooth, occasionally with small oil drops, IKI–, weakly CB+, (4.5–)4.7–6 × 1.3–2 μm, *L* = 5.44 μm, *W* = 1.76 μm, *Q* = 2.89–2.93 (*n* = 60/2).

*Notes*. — Phylogenetically, *Cyanosporus tenuis* grouped together with *C. arbuti* (Spirin) B.K. Cui & Shun Liu, *C. luteocaesius* (A. David) B.K. Cui, L.L. Shen & Y.C. Dai and *C. simulans* (P. Karst.) B.K. Cui & Shun Liu. Morphologically, *C. arbuti* differs in having narrower hyphae in the context and smaller basidiospores (4.1–5.1 × 1–1.2 μm) (Table 2); *C. luteocaesius* differs in larger pores (3–5 per mm; Table 2); *C. simulans* differs in smaller basidia (10–14.8 × 3.7–5.2 μm; Table 2). *Cyanosporus fusiformis*, *C. piceicola*, *C. tricolor* B.K. Cui, L.L. Shen & Y.C. Dai and *C. ungulatus* are also distributed in Sichuan Province, but *C. fusiformis* has a semicircular pileus, fusiform cystidioles presenting in the hymenium and narrow allantoid basidiospores (4.5–5.2 × 0.8–1.1 μm) (Table 2); *C. piceicola* has sub-rotund pileus, small angular pores, and slightly thick-walled and allantoid basidiospores (4–4.5 × 0.9–1.3 μm) (Table 2); *C. tricolor* has a white, blue, and pale mouse gray pileal surface when fresh (Shen et al., 2019); *C. ungulatus* has ungulate basidiocarps (Shen et al., 2019).

Additional specimen (paratype) examined. **CHINA**. Sichuan Province, Puge County, Luoji Mountain, on fallen trunk of *Picea*, 19 September 2012, *Dai 12974* (BJFC).

***Cyanosporus arbuti*** (Spirin) B.K. Cui & Shun Liu, **comb. nov.**

MycoBank: MB 838422

Basionym: *Postia arbuti* Spirin, Fungal Systematics and Evolution 1: 113, 2018.

*Notes*. — *Postia arbuti* was described from the United States (Miettinen et al., 2018). It is characterized by conchate, pendant to effused-reflexed basidiocarps with almost glabrous to matt pileal surfaces. It usually grows on *Arbutus menziesii* and is widely distributed in temperate areas of North America (North-West). In our study, the phylogenetic analysis (Figures 1–3) strongly supports its placement in *Cyanosporus*. For a detailed description of *Postia arbuti*, see Miettinen et al. (2018).

***Cyanosporus auricoma*** (Spirin & Niemelä) B.K. Cui & Shun Liu, **comb. nov.**

MycoBank: MB 838423

Basionym: *Postia auricoma* Spirin & Niemelä, Fungal Systematics and Evolution 1: 115, 2018.

= *Cyanosporus mongolicus* B.K. Cui, L.L. Shen & Y.C. Dai, Persoonia 42: 115, 2019.

*Notes*. — *Postia auricoma* was described by Miettinen et al. (2018) and *Cyanosporus mongolicus* was described by Shen et al. (2019). Phylogenetically, the two species formed a highly supported lineage (Figures 1–3) and as the morphological characters of *C. mongolicus* fit well with *Postia auricoma*, we treat *C. mongolicus* as a synonym of *P. auricoma*, and *P. auricoma* is transferred to *Cyanosporus* as a new combination.

Specimens examined: **CHINA**. Inner Mongolia, Ewenk, Honghuaerji Nature Reserve, on fallen trunk of *Pinus*, 19 October 2015, *Cui 13518*, *13519* (BJFC).

***Cyanosporus bifaria*** (Spirin) B.K. Cui & Shun Liu, **comb. nov.**

MycoBank: MB 838424

Basionym: *Postia bifaria* Spirin, Fungal Systematics and Evolution 1: 115, 2018.

*Notes*. — *Postia bifaria* was described by Miettinen et al. (2018). The morphological characters of the two specimens from China fit well with *Postia bifaria* and phylogenetically they clustered together within the genus *Cyanosporus*. Based on morphological characters and phylogenetic analysis, we transferred *Postia bifaria* to *Cyanosporus* as a new combination. For a detailed description of *Postia bifaria*, see Miettinen et al. (2018).

Specimens examined: **CHINA**. Sichuan Province, Xiangcheng County, on fallen trunk of *Pinus*, 12 August 2019, *Cui 17445* (BJFC); Zhaojue County, on stump of *Pinus*, 16 September 2019, *Cui 17806* (BJFC).

***Cyanosporus caesiosimulans*** (G.F. Atk.) B.K. Cui & Shun Liu, **comb. nov.**

MycoBank: MB 838425

Basionym: *Tyromyces caesiosimulan*s G.F. Atk., Annales Mycologici 6: 61, 1908.

= *Postia caesiosimulans* (G.F. Atk.) Spirin & Miettinen, Fungal Systematics and Evolution 1: 117, 2018.

*Notes*. — *Tyromyces caesiosimulans* was introduced primarily based on its globose basidiospores (Atkinson, 1908). Miettinen et al. (2018) transferred it to the *Postia caesia* complex based on morphological and molecular evidence. In our study, the phylogenetic analysis strongly supports its placement in *Cyanosporus* (Figures 1–3). For a detailed description of *Postia caesiosimulans*, see Miettinen et al. (2018).

***Cyanosporus coeruleivirens*** (Corner) B.K. Cui, Shun Liu & Y.C. Dai, **comb. nov.**

MycoBank: MB 838426

Basionym: *Tyromyces coeruleivirens* Corner, Beihefte zur Nova Hedwigia 96: 163, 1989.

= *Postia coeruleivirens* (Corner) V. Papp, Mycotaxon 129 (2): 411, 2015.

*Notes*. — *Tyromyces coeruleivirens* was described from Borneo (Corner, 1989). It has a greenish pileus, a monomitic hyphal system, and allantoid inamyloid basidiospores (Corner, 1989; Hattori, 2002), and was considered as a member of the *Postia caesia* group (Hattori, 2002). Papp (2014) transferred *Tyromyces coeruleivirens* to the *Postia caesia* complex as a new combination. In our study, *Postia coeruleivirens* is transferred to *Cyanosporus* as a new combination; phylogenetically it is closely related to *C. subhirsutus* (Figures 1–3). Morphologically, both *C. coeruleivirens* and *C. subhirsutus* have annual basidiocarps, a monomitic hyphal system and allantoid basidiospores, but *C. subhirsutus* differs in having a disc-shaped pileus, hirsute and zonate pileal surface and larger pores (2–3 per mm; Shen et al., 2019). For a detailed description of *Postia coeruleivirens*, see Miettinen et al. (2018).

Specimens examined: **CHINA**. Hunan Province, Changde, Hefu Forest Park, on fallen angiosperm trunk, 17 October 2018, *Dai 19220* (BJFC); Zhejiang Province, Hangzhou, Jiuxi Forest Park, on fallen angiosperm trunk, 17 October 2010, *Dai 11834* (BJFC).

***Cyanosporus comata*** (Miettinen) B.K. Cui & Shun Liu, **comb. nov.**

MycoBank: MB 838427

Basionym: *Postia comata* Miettinen, Fungal Systematics and Evolution 1: 118, 2018.

*Notes*. — *Postia comata* was described from the United States (Miettinen et al., 2018). It is morphologically similar to *P. livens* Miettinen & Vlasák but differs in having mostly thick-walled tramal hyphae and slightly smaller basidiospores (Miettinen et al., 2018). Its closest relative is the East Asian *P. bifaria*, which has smaller pores (6–8 per mm) and basidiospores (3.7–4.4 × 1.0–1.2 μm), and thin-walled tramal hyphae that collapse (Miettinen et al., 2018). *Postia comata* is transferred to *Cyanosporus* as a new combination based on morphological characters and molecular phylogeny. For a detailed description of *Postia comata*, see Miettinen et al. (2018).

Specimens examined: **CHINA**. Sichuan Province, Jiuzhaigou County, Zhangzha, on stump of *Picea*, 19 September 2020, *Cui 18546* (BJFC); Xizang (Tibet), Mangkang County, on stump of *Abies*, 8 September 2020, *Cui 18388* (BJFC).

***Cyanosporus cyanescens*** (Miettinen) B.K. Cui & Shun Liu, **comb. nov.**

MycoBank: MB 838428

Basionym: *Postia cyanescens* Miettinen, Fungal Systematics and Evolution 1: 119, 2018.

*Notes*. — *Postia cyanescens* was described by Miettinen et al. (2018). This species is characterized by thin, conchate to flabelliform basidiocarps with light bluish grayish tinted pore surfaces, and long and narrow basidiospores (4.7–6.1 × 1.1–1.6 μm). In our study, the phylogenetic analysis (Figures 1–3) strongly supports its placement in *Cyanosporus*, so we transferred *P. cyanescens* to *Cyanosporus* as a new combination. For a detailed description of *Postia cyanescens*, see Miettinen et al. (2018).

***Cyanosporus glauca*** (Spirin & Miettinen) B.K. Cui & Shun Liu, **comb. nov.**

MycoBank: MB 838429

Basionym: *Postia glauca* Spirin & Miettinen, Fungal Systematics and Evolution 1: 120, 2018.

*Notes*. — *Postia glauca* was described by Miettinen et al. (2018), it is characterized by thin, small and conchate basidiocarps with plumbeous to bluish gray or grayish brown pileal surfaces, and cream to light bluish grayish pore surfaces. In our study, the phylogenetic analysis (Figures 1–3) supports its placement in *Cyanosporus*, so we transferred it to *Cyanosporus* as a new combination. For a detailed description of *Postia glauca*, see Miettinen et al. (2018).

***Cyanosporus gossypina*** (Moug. & Lév.) B.K. Cui & Shun Liu, **comb. nov.**

MycoBank: MB 838430

Basionym: *Polyporus gossypinus* Moug. & Lév., Annales des Sciences Naturelles Botanique 9: 124, 1848.

= *Postia gossypina* (Moug. & Lév.) Spirin & B. Rivoire, Fungal Systematics and Evolution 1: 120, 2018.

*Notes*. — *Postia gossypina* was proposed by Miettinen et al. (2018). Based on morphological characters and phylogenetic analysis, we transferred *P. gossypina* to *Cyanosporus* as a new combination. For a detailed description of *Postia gossypina*, see Miettinen et al. (2018).

***Cyanosporus livens*** (Miettinen & Vlasák) B.K. Cui & Shun Liu, **comb. nov.**

MycoBank: MB 838431

Basionym: *Postia livens* Miettinen & Vlasák, Fungal Systematics and Evolution 1: 120, 2018.

*Notes*. — *Postia livens* was described from North America. It is the most common representative of the *P. caesia* complex in North America, and it can be easily identified by its plumbeous to bluish gray to ochraceous pileal surface often with bluish tints (Miettinen et al., 2018). In our study, it grouped with other species of the *P. caesia* complex in *Cyanosporus* (Figures 1–3), so, we transferred it into *Cyanosporus* as a new combination. For a detailed description of *Postia livens*, see Miettinen et al. (2018).

***Cyanosporus magna*** (Miettinen) B.K. Cui & Shun Liu, **comb. nov.**

MycoBank: MB 838432

Basionym: *Postia magna* Miettinen, Fungal Systematics and Evolution 1: 121, 2018.

*Notes*. — *Postia magna* was described from China (Miettinen et al., 2018). Although we did not find the type specimen, we have examined two specimens from China, and its morphological characters fit well with this species. The phylogenetic analysis (Figures 1–3) also strongly supports its placement in *Cyanosporus*. So, we transferred *Postia magna* to *Cyanosporus* as a new combination. For a detailed description of *Postia magna*, see Miettinen et al. (2018).

Specimens examined: **CHINA**. Hainan Province, Ledong County, Jianfengling Nature Reserve, on fallen angiosperm trunk, 12 May 2009, Cui 10854 (BJFC); Jilin Province, Fusong County, Lushuihe Forest Park, on fallen angiosperm branch, 11 August 2011, *Cui 10094* (BJFC); Yunnan Province, Binchuan County, Jizu Mountain, on fallen angiosperm branch, 14 September 2018, *Cui 16983* (BJFC).

***Cyanosporus populi*** (Miettinen) B.K. Cui & Shun Liu, **comb. nov.**

MycoBank: MB 838433

Basionym: *Postia populi* Miettinen, Fungal Systematics and Evolution 1: 122, 2018.

*Notes*. — *Postia populi* was described by Miettinen et al. (2018). Based on morphological characters and phylogenetic analyses, we transferred *P. populi* to *Cyanosporus* as a new combination. For a detailed description of *Postia populi*, see Miettinen et al. (2018).

Specimens examined: **CHINA**. Sichuan Province, Muli County, on fallen trunk of *Pinus*, 16 August 2019, *Cui 17549* (BJFC); Yunnan Province, Lijiang, Yulong Snow Mountain, on fallen trunk of *Picea*, 16 September 2018, *Cui 17087a* (BJFC).

***Cyanosporus simulans*** (P. Karst.) B.K. Cui & Shun Liu, **comb. nov.**

MycoBank: MB 838434

Basionym: *Bjerkandera simulans* P. Karst., Revue Mycologique Toulouse 10: 73, 1888.

= *Postia simulans* (P. Karst.) Spirin & B. Rivoire, Fungal Systematics and Evolution 1: 123, 2018.

*Notes*. — This species was described by Karsten (1888) as *Bjerkandera simulans*. Recently, *Bjerkandera simulans* was treated as an independent species in the *Postia caesia* complex by Miettinen et al. (2018). In our study, the phylogenetic analysis (Figures 1–3) supports its placement in *Cyanosporus*. For a detailed description of *Postia simulans*, see Miettinen et al. (2018).

***Cyanosporus subviridis*** (Ryvarden & Guzmán) B.K. Cui & Shun Liu, **comb. nov.**

MycoBank: MB 838435

Basionym: *Tyromyces subviridis* Ryvarden & Guzmán, Mycotaxon 78: 252, 2001.

= *Postia subviridis* (Ryvarden & Guzmán) Spirin, Fungal Systematics and Evolution 1: 125, 2018.

*Notes*. — *Tyromyces subviridis* was described from a highland conifer forest in Mexico (Guzmán and Ryvarden, 2001). Miettinen et al. (2018) studied the type specimen, and transferred it to the *Postia caesia* complex. In our study, the phylogenetic analysis (Figures 1–3) supports its placement in *Cyanosporus*. For a detailed description of *Postia subviridis*, see Miettinen et al. (2018).

***Cyanosporus yanae*** (Miettinen & Kotiranta) B.K. Cui & Shun Liu, **comb. nov.**

MycoBank: MB 838436

Basionym: *Postia yanae* Miettinen & Kotir., Fungal Systematics and Evolution 1: 125, 2018.

*Notes*. — *Postia yanae* was described by Miettinen et al. (2018). It is characterized by effused-reflexed or resupinate basidiocarps with detached or adnate margins, and white, with light to strong bluish grayish tinted pore surfaces. In our study, the phylogenetic analysis (Figures 1–3) supports its placement in *Cyanosporus*. For a detailed description of *Postia yanae*, see Miettinen et al. (2018).

### Other Specimens of *Cyanosporus* Examined

*Cyanosporus alni* (Niemelä & Vampola) B.K. Cui, L.L. Shen & Y.C. Dai. **CHINA**. Guizhou Province, Suiyang County, Kuankuoshui Nature Reserve, on fallen angiosperm trunk, 26 June 2014, *Dai 15060* (BJFC); Hebei Province, Xinglong County, Wulingshan Nature Reserve, on fallen angiosperm trunk, 29 August 2009, Cui 7185 (BJFC). **CZECH REPUBLIC**. Ceske Budejovice, on fallen trunk of *Fagus*, 22 November 2011, *Dai 12709* (BJFC). **FINLAND**. Uusimaa, Vantaa, Tamisto Nature Reserve, on fallen trunk of *Populus*, 4 November 2011, *Dai 12641* (BJFC). **POLAND**. Brynica, Mcrow, on fallen trunk of *Fagus*, 3 October 2014, *Dai 14845* (BJFC).

*Cyanosporus caesius*. **FINLAND**. Uusimaa, Vantaa, Tamisto Nature Reserve, on fallen trunk of *Picea*, 3 November 2011, *Dai 12605* (BJFC). **SPAIN**. Cadiz Province, Sierra Grazalema Natural Park, on fallen trunk of Abies, 22 November 2005, Dai 7438 (BJFC).

*Cyanosporus fusiformis*. **CHINA**. Guizhou Province, Suiyang County, Kuankuoshui Nature Reserve, on dead angiosperm tree, 26 November 2014, *Dai 15036* (BJFC, holotype); Sichuan Province, Luding County, Hailuogou Forest Park, on dead tree of *Rhododendron*, 20 October 2012, *Cui 10775* (BJFC, paratype).

*Cyanosporus microporus*. **CHINA**. Yunnan Province, Pu’er, Taiyanghe National Forest Park, on fallen angiosperm trunk, 8 July 2013, *Cui 11014* (BJFC, holotype); Chuxiong, Zixishan Nature Reserve, on dead angiosperm tree, 28 August 2010, *Dai 11717* (BJFC, paratype).

*Cyanosporus piceicola*. **CHINA**. Sichuan Province, Jiuzhaigou County, Jiuzhaigou Nature Reserve, on stump of *Picea*, 11 October 2012, *Cui 10626* (BJFC, holotype), on fallen trunk of *Picea*, 11 October 2012, *Cui 10617* (BJFC, paratype); Xizang (Tibet), Linzhi, Sejila Mountain, on fallen trunk of *Picea*, 18 September 2014, *Cui 12158* (BJFC, paratype); Milin County, Nanyigou Forest Park, on fallen trunk of *Picea*, 16 September 2014, *Cui 12088* (BJFC, paratype); Yunnan Province, Weixi County, Laojunshan Nature Reserve, on fallen trunk of *Picea*, 21 September 2011, *Cui 10446* (BJFC, paratype).

*Cyanosporus subhirsutus*. **CHINA**. Fujian Province, Yongjing County, Huboliao Nature Reserve, on fallen angiosperm branch, 26 October 2013, *Cui 11330* (BJFC, paratype); Guizhou Province, Jiangkou County, Fanjingshan Nature Reserve, on fallen trunk of *Pterocarya*, 21 November 2014, *Dai 14892* (BJFC, holotype); Yunnan Province, Pu’er, Taiyanghe National Forest Park, on fallen angiosperm trunk, 8 July 2013, *Cui 11019* (BJFC, paratype).

*Cyanosporus subcaesius*. **FINLAND**. Helsinki, Arabia, on angiosperm stump, 23 November 1996, Dai 2345 (IFP); Vantaa, on fallen trunk of *Prunus*, 4 October 1997, Dai 2725 (IFP).

*Cyanosporus tricolor*. **CHINA**. Sichuan Province, Luding County, Hailuogou Forest Park, on fallen trunk of *Abies*, 20 October 2012, *Cui 10790* (BJFC, paratype), on fallen trunk of *Picea*, 20 October 2012, *Cui 10780* (BJFC, paratype); Xizang (Tibet), Motuo County, on fallen branch of *Abies*, 20 September 2014, *Cui 12233* (BJFC, holotype).

*Cyanosporus ungulatus*. **CHINA**. Sichuan Province, Mianning County, Lingshansi Park, on fallen branch of *Castanopsis*, 17 September 2012, *Dai 12897* (BJFC, holotype); Luding County, Hailuogou Forest Park, on fallen trunk of *Abies*, 20 October 2012, *Cui 10778* (BJFC, paratype).

## Discussion

The genus *Cyanosporus*, usually with blue-tinted basidiocarps, is easy to recognize, but identification to species level is difficult as morphological features are quite similar among the species. The main morphological characters of the species in *Cyanosporus* are provided in Table 2.

In our current phylogenetic analyses, the genus *Cyanosporus* is supported as an independent genus; 31 species grouped together and formed a highly supported lineage (100% BS, 100% MP, 1.00 BPP; Figures 1–3), and were distant from other genera of *Postia* sensu lato. The current study also confirmed that *Cyanosporus* belongs to the antrodia clade phylogenetically and clusters with other brown-rot fungal genera, such as *Amaropostia* B.K. Cui, L.L. Shen & Y.C. Dai, *Amylocystis* Bondartsev & Singer ex Singer, *Calcipostia* B.K. Cui, L.L. Shen & Y.C. Dai, *Cystidiopostia* B.K. Cui, L.L. Shen & Y.C. Dai, *Fuscopostia* B.K. Cui, L.L. Shen & Y.C. Dai, *Oligoporus* Bref., *Osteina* Donk, *Postia* Fr. and *Spongiporus* Murrill, which is similar to previous studies (Ortiz-Santana et al., 2013; Ryvarden and Melo, 2014; Han et al., 2016; Shen et al., 2019).

The nomenclatural and taxonomic history of the *Postia caesia* complex was critically reviewed by Papp (2014), the generic name of the *P. caesia* complex have been changed many times, Papp proposed *Cyanosporus* as a subgenus of *Postia* to contain the *P. caesia* complex. Miettinen et al. (2018) revised the species concept of the *Postia caesia* complex based on morphology and two gene markers (ITS and TEF) and discussed that TEF sequences are more reliable for molecular identification of the *P. caesia* complex than ITS sequences. They also indicated that host tree is important for species identification of the *P. caesia* complex; their study raised the species number of the *Postia caesia* complex from 10 to 24, but their study did not focus on the taxonomic status for the *P. caesia* complex of *Postia* sensu lato.

Previously, species identification of the *Postia caesia* complex was only based on morphological characters and host trees in China. Samples grown on angiosperm woods were usually identified as *Postia alni* Niemelä & Vampola and those on gymnosperm woods as *Postia caesia*; only two species were recorded from China before Dai (2012). Shen et al. (2019) carried out a comprehensive phylogenetic and taxonomic study of *Postia* and related genera based on morphological characters and the combined seven-gene (ITS + nLSU + nSSU + mtSSU + RPB1 + RPB2 + TEF) sequences, *Cyanosporus* was confirmed as an independent genus of *Postia* sensu lato and 12 species belonging to the *P. caesia* complex were recognized in *Cyanosporus*, which was distant from *Postia* s. s.

In the current study, the novel species were supported by phylogenetic analyses based on ITS + TEF sequences, ITS + nLSU + TEF sequences and ITS + nLSU + nSSU + mtSSU + RPB1 + RPB2 + TEF sequences, respectively, but for the ITS sequences, several species have very similar base pairs and could not be separated by ITS sequences. The suitable DNA barcoding gene is the TEF gene for species identification of *Cyanosporus*, this result is consistent with a previous study by Miettinen et al. (2018). Our study expanded the number of *Cyanosporus* species to 31 around the world including 19 species from China. Our study indicated that more cryptic species could be discovered by combined evidence of morphological characters, molecular data, host trees and distribution areas in species complexes. However, several species of the *Postia caesia* complex, such as, *P. africana* (Ryvarden) V. Papp, *P. amyloidea* (Corner) V. Papp, *P. atrostrigosa* (Cooke) Rajchenb. and *P. caesioflava* (Pat.) V. Papp, are not included in the current phylogenetic analysis due to the lack of DNA sequences. Although the morphological characters of these species fit well with *Cyanosporus*, we did not transfer them to *Cyanosporus* in the current study. A fully resolved phylogeny for *Cyanosporus* and its related genera requires evolutionary information from more samples and more conserved gene markers.

In addition, some species with yellow basidiocarps, such as *C. auricoma*, *C. caesioflava*, and *C. luteocaesius*, and some species with white to cream colored basidiocarps, such as *C. bubalinus* and *C. nothofagicola*, are grouped together within *Cyanosporus*; this expanded the concept of the genus to include taxa without blue-tinted basidiocarps. More novel species might be discovered from different regions in future studies.

## Data Availability Statement

The datasets presented in this study can be found in online repositories. The names of the repository/repositories and accession number(s) can be found below: http://purl.org/phylo/treebase, 27274.

## Author Contributions

B-KC and SL designed the experiments. SL, L-LS, GG, and B-KC prepared the samples. SL, YW, and T-MX conducted the molecular experiments and analyzed the data. SL, GG, and B-KC drafted the manuscript. All the authors approved the manuscript.

## Conflict of Interest

The authors declare that the research was conducted in the absence of any commercial or financial relationships that could be construed as a potential conflict of interest.

## Footnotes

1 http://mafft.cbrc.jp/alignment/server/

2 http://www.treebase.org

3 http://tree.bio.ed.ac.uk/software/figtree/

## Nomenclature

AR: Argentina; BI: Bayesian inference; BJFC: Herbarium of the Institute of Microbiology, Beijing Forestry University; BGI: Beijing Genomics Institute; BPP: Bayesian posterior probabilities; BT: Bootstrap; CB: Cotton Blue; CB +: cyanophilous; CB–: acyanophilous; GB: Great Britain; GTR + I + G: General time reversible + proportion invariant + gamma; CTAB: Cetyl trimethylammonium bromide; IFP: Herbarium of the Institute of Applied Ecology, Chinese Academy of Sciences; IKI: Melzer’s reagent, IKI–: neither amyloid nor dextrinoid; ILD: Incongruence length difference test; ITS: Internal transcribed spacer; KOH: 5% potassium hydroxide; L: mean spore length (arithmetic average of all spores); ML: Maximum likelihood; MP: Maximum parsimony; MPT: Most Parsimonious Tree; mtSSU: Mitochondrial small subunit rRNA; n (a/b): number of spores (a) measured from given number (b) of specimens; nLSU: Nuclear large subunit rDNA; nSSU: Nuclear small subunit rRNA; Q: variation in the L/W ratios between the specimens studied; RPB1: DNA-directed RNA polymerase II subunit 1; RPB2: DNA-directed RNA polymerase II subunit 2; TL: Tree length; W: mean spore width (arithmetic average of all spores); CI: Consistency Index; RI: Retention Index; RC: Rescaled Consistency Index; HI: Homoplasy Index; TEF: Translation elongation factor 1-α.
